# Saturation Transfer Difference NMR and Molecular Docking Interaction Study of Aralkyl-Thiodigalactosides as Potential Inhibitors of the Human-Galectin-3 Protein

**DOI:** 10.3390/ijms25031742

**Published:** 2024-02-01

**Authors:** Fanni Hőgye, László Bence Farkas, Álex Kálmán Balogh, László Szilágyi, Samar Alnukari, István Bajza, Anikó Borbás, Krisztina Fehér, Tünde Zita Illyés, István Timári

**Affiliations:** 1 Department of Organic Chemistry, University of Debrecen, Egyetem tér 1, H-4032 Debrecen, Hungary; hogye.fanni@science.unideb.hu (F.H.); farkas.laszlo.bence@science.unideb.hu (L.B.F.); szilagyi.laszlo@science.unideb.hu (L.S.); 2 HUN-REN-UD Molecular Recognition and Interaction Research Group, University of Debrecen, Egyetem tér 1, H-4032 Debrecen, Hungary; balogh.alex@science.unideb.hu (Á.K.B.); samar.alnukari@science.unideb.hu (S.A.); borbas.aniko@science.unideb.hu (A.B.); feher.krisztina@science.unideb.hu (K.F.); 3 GlycOptim Kft., Egyetem tér 1, H-4032 Debrecen, Hungary; istvan.bajza@gmail.hu; 4 Department of Pharmaceutical Chemistry, University of Debrecen, Egyetem tér 1, H-4032 Debrecen, Hungary

**Keywords:** lectin, galectin-3, thiodigalactosides, NMR spectroscopy, STD NMR, molecular docking

## Abstract

Human Galectin-3 (*h*Gal-3) is a protein that selectively binds to β-galactosides and holds diverse roles in both normal and pathological circumstances. Therefore, targeting *h*Gal-3 has become a vibrant area of research in the pharmaceutical chemistry. As a step towards the development of novel *h*Gal-3 inhibitors, we synthesized and investigated derivatives of thiodigalactoside (TDG) modified with different aromatic substituents. Specifically, we describe a high-yielding synthetic route of thiodigalactoside (TDG); an optimized procedure for the synthesis of the novel 3,3′-di-*O*-(quinoline-2-yl)methyl)-TDG and three other known, symmetric 3,3′-di-*O*-TDG derivatives ((naphthalene-2yl)methyl, benzyl, (7-methoxy-2H-1-benzopyran-2-on-4-yl)methyl). In the present study, using competition Saturation Transfer Difference (STD) NMR spectroscopy, we determined the dissociation constant (K_d_) of the former three TDG derivatives produced to characterize the strength of the interaction with the target protein (*h*Gal-3). Based on the K_d_ values determined, the (naphthalen-2-yl)methyl, the (quinolin-2-yl)methyl and the benzyl derivatives bind to *h*Gal-3 94, 30 and 24 times more strongly than TDG. Then, we studied the binding modes of the derivatives in silico by molecular docking calculations. Docking poses similar to the canonical binding modes of well-known *h*Gal-3 inhibitors have been found. However, additional binding forces, cation–π interactions between the arginine residues in the binding pocket of the protein and the aromatic groups of the ligands, have been established as significant features. Our results offer a molecular-level understanding of the varying affinities observed among the synthesized thiodigalactoside derivatives, which can be a key aspect in the future development of more effective ligands of *h*Gal-3.

## 1. Introduction

Human galectin-3 (*h*Gal-3), a galactose-binding lectin, is implicated in numerous physiological and pathological processes, such as inflammation, fibrosis, heart disease, tumor progression and stroke [1,2,3,4,5,6]. Being a potential therapeutic target, a great deal of attention has been directed toward developing *h*Gal-3 inhibitors, including various glycoconjugates and glycomimetics [7]. The 4-OH and 6-OH groups of galactose are essential for their binding to *h*Gal-3, while the 2-OH and 3-OH groups do not directly interact with this lectin. Therefore, research into the development of galactoside-based high-affinity *h*Gal-3 antagonists has focused on chemical modifications at the C-3 position, as the C-3 group of galactose is well positioned to fit into the ligand groove of the carbohydrate recognition domain of Gal-3 [8]. Among the best Gal-3 ligands developed so far are 3,3′-*O/N*-di-aryl/aralkyl substituted β-thiodigalactoside (TDG) derivatives [9,10,11]. It was shown that the aromatic groups at the C-3 position of TDG increase the affinity for *h*Gal-3 due to π-electron stacking and the accompanying favorable interactions, resulting in an extremely strong (nanomolar) galectin–glycomimetic interaction. Another advantage is that TDG derivatives are resistant to enzymatic degradation in vivo due to their thioglycosidic bond [12]. Nilsson and co-workers developed several symmetrically 3,3′-*N*-disubstituted thiodigalactosides, some of which were shown to bind to *h*Gal-3 with nanomolar affinity [9,10]. In the synthesis leading to these 3,3′-diaza-TDG derivatives, tri-isopropylsilyl thiogalactoside was used as a masked glycosyl thiol nucleophile, which was reacted with glycosyl bromide as an electrophilic acceptor in the presence of tetrabutylammonium fluoride (TBAF) [13]. In an alternative synthesis method, galactopyranosyl isothiuronium salt was applied as a sulfur-bearing nucleophile in the reaction with the glycosyl bromide electrophile [13]. Bojarova, P. and her co-workers synthesized 3,3′-*O*-disubstituted thiodigalactosides directly from the commercially available TDG in a single step, using stannylidene-mediated regioselective benzylations with readily available bromides [11]. The reaction of TDG with Bu_2_SnO in the presence of tetra-*n*-butyl ammonium bromide and *N,N*-diisopropylethylamine afforded a tin intermediate [14], which was reacted with the respective bromides in a one-pot reaction to yield the desired compounds [11]. We have also synthesized several sulfur- and selenium-containing carbohydrate derivatives and tested their inhibitory potencies against *h*Gal-3 [15,16]. Detailed structural investigation of the binding of some selenoglycosides to *h*Gal-3 has been recently performed by NMR spectroscopy, including improved ^77^Se NMR-based methods, X-ray crystallography and molecular dynamics (MD) simulations [17,18,19].

NMR spectroscopy is one of the widely used experimental techniques for studying molecular interactions in atomic details [20,21,22,23,24]. The Saturation Transfer Difference (STD) NMR method can be applied best for investigating the ligand–protein interaction of moderate- to weak-affinity ligands (K_D_ = 10^−6^–10^−3^ M) characterized by fast ligand exchange [25,26]. In the STD experiment, the resonance signals of the bound ligand appear in the NMR spectrum, while signals of non-binding ligands do not. It is also utilized for epitope mapping of binding ligands [27]. The binding mode of TDG has been investigated in detail with multiple biophysical methods, including high-resolution X-ray crystallography (PDB code: 4JC1). The carbohydrate binding domain of *h*Gal-3 is made up of two β-sheets with six strands named S1–S6, and the binding site is located at strands from S2 to S6 [28]. The canonical binding mode of TDG involves a stacking interaction of the inner/proximal galactose ring with the aromatic ring of TRP-181, while the distal ring is located farther away from the binding site. Molecular docking is a computational method of choice for gaining insight into the interactions of small molecules with their macromolecular targets [29].

**Scheme 1:**
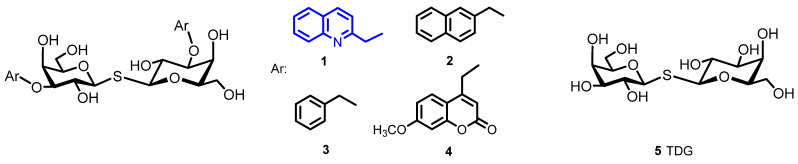
Structures of the synthesized and investigated thiodigalactosides (**1**–**4**) and TDG (**5**). The new compound is indicated by blue color.

Here, we report the synthesis of a novel 3,3′-(quinoline-2-yl)methyl-di-*O*-disubstituted TDG derivative (**1**, Scheme 1) and the study of its interaction with *h*Gal-3 using STD NMR spectroscopy and molecular docking simulations. We chose the quinoline as a structural motif to incorporate into our newly synthesized compound because it has been found in the structure of many bioactive molecules and is often applied for drug design in medicinal chemistry [30,31,32,33,34]. To compare the binding properties of the novel derivative to *h*Gal-3, three known 3,3′-aralkyl-disubstituted thiodigalactosides described previously [11], namely, naphthalene-2-yl)methyl (**2**), benzyl (**3**) and (7-methoxy-2H-1-benzopyran-2-on-4-yl)methyl (**4**) derivatives at Scheme 1, were also synthesized using modified synthetic strategies for their preparation.

## 2. Results and Discussion

### 2.1. Chemical Synthesis

**Scheme 2:**
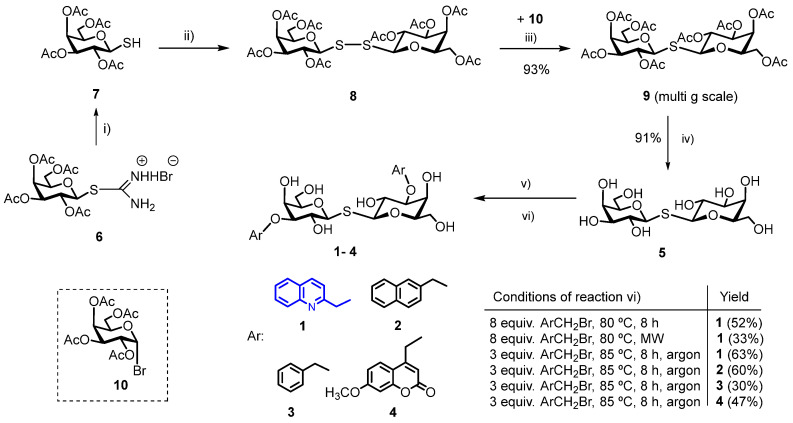
Synthetic route of TDG at multi-gram scales and of 3,3′-*O*-aralkyl disubstituted symmetrical thiodigalactosides **1**–**4** starting from TDG using dibutyltin(IV) oxide and aralkylbromides. (i) Na_2_S_2_O_5_/H_2_O/CH_2_Cl_2_ 3 h reflux, for thiol formation [38]; (ii) H_2_O_2_/NaI/EtOAc, rt, 1 h, for disulfide formation [39]; (iii) 1-Br-2,3,4,6-tetra-*O*-acetyl-α-D-galactopyranose (**10**), CH_3_CN, NaBH_4_, argon atmosphere, rt, 24 h [35]; (iv) dry CH_3_OH/CH_3_ONa, rt, 1 h; (v) Bu_2_SnO/MeOH, reflux, 3 h; (vi) optimized conditions: 3 equiv. ArCH_2_Br, TBAB, dry 1,4-dioxane, argon atmosphere, 85 °C, 8 h [11,14]. The new compound is indicated by blue color.

In this work, we have synthesized a new *N*-heterocyclic derivative of TDG, bis-{3-*O*-[(quinolin-2-yl)methyl]-β-D-galactopyranosyl}-sulfane (**1**) starting from TDG, in two steps in good yield (Scheme 2). We have also developed a reproducible, inexpensive, scale-up synthetic route to produce thiodigalactoside (TDG, **5**), based on a modified synthetic method described in the literature [35]. For this, bis-(2,3,4,6-tetra-*O*-acetyl-β-D-galactopyranosyl)-disulfide (**8**) was synthesized on a multi-gram scale in high yield from the appropriate thiol (**7**) using H_2_O_2_ and a catalytic amount of NaI in ethyl acetate at room temperature [36]. Reaction of bis-(2,3,4,6-tetra-*O*-acetyl-β-D-galactopyranosyl)-disulfide with two equivalents of 1-bromo-2,3,4,6-tetra-*O*-acetyl-α-D-galactopyranose (**10**) and six equivalents of NaBH_4_ under an argon atmosphere produced acetyl-protected TDG (**9**) as a white powder in excellent yield (87%) in 12 h. Deprotection by the Zemplén method afforded TDG (**5**) in high yield (91%).

Bojarova, P. and her coworkers optimized the reaction conditions with their pilot compound, 3,3′-*O*-dibenzyl substituted thiodigalactoside (TDG), in a one-pot reaction [11]. They used the MW-assisted Sn-acetal-mediated regioselective substitution of TDG with a large excess of the benzylation reagent (8 eq) in 1,4-dry dioxane, at 90 °C. The yield of the reaction was 36%. An expensive reagent always justifies the use of reduced reagent excess when scaling up the reactions. In order to synthesize the new bis-{3-*O*-[(quinoline-2-yl)methyl]-β-D-galactopyranosyl}-sulfane (**1**), we optimized the conditions of the Sn-mediated regioselective alkylation reaction. In the first step, our aim was to synthesize the TDG stannylidene-acetal started from TDG with dibutyltin(IV) oxide (Bu_2_SnO) in methanol at reflux temperature, and in the second step, to react the acetal with varying reagent excess in dry 1,4-dioxane in the presence of tetrabutylammonium bromide (TBAB). The test reactions were carried out with 0.05 g of TDG (**5**) under different conditions. In the first experiment, compound **1** was formed in 52% at 80 °C using eight equivalents of 2-(bromomethyl)quinoline for TDG. MW-assisted reaction with eight equivalents of reagent at the same temperature (80 °C) yielded the desired compound **1** in 33%. In the last experiment, when three equivalents of reagent were used at 85 °C, the expected compound was formed in 63%. In this case, we used an argon atmosphere. Based on these results, further syntheses were performed with three equivalents of bromide reagent for TDG.

In order to compare binding properties of the new bis-{3-*O*-[(quinolin-2-yl)methyl]-β-D-galactopyranosyl}-sulfane (**1**) to *h*Gal-3, three other known 3,3′-disubstituted-*O*-aralkyl thiodigalactoside derivatives [11,37] were also synthesized from TDG (**2**–**4**) under the optimized conditions (three equivalents of bromide, 1,4-dry dioxane, TBAB, 85 °C), as shown in Scheme 2. The preparation of stannylidene-acetal was performed at multi-gram scale; the yields of TDG-derivatives were ranging from 30% (for benzyl derivative, **3**) to 63% [(quinolin-2-yl)methyl derivative **1**]. The structure and interaction of the synthesized compounds with *h*Gal-3 were characterized by ^1^H STD NMR experiments and molecular docking simulations, as described in the following sections.

### 2.2. Determination of the Equilibrium Dissociation Constants (K_D_) for Aromatic Thiodigalactoside Derivatives Bound to hGal-3 by Competition STD ^1^H NMR Method

The competition STD NMR method provides an opportunity to characterize moderately and strongly bound protein–ligand systems (K_D_ = 10^−6^ M − 10^−9^ M), where the determination is not feasible in direct STD experiment due to kinetic reasons [40,41]. In the competition experiment, the concentration of the target protein and the reference ligand is kept at a constant level. An STD spectrum of the starting sample state is obtained. Then, by adding the competitive ligand in increasing concentration step-by-step, the resonance signal changes in the STD spectra can be monitored. If the competitor ligand binds strongly to the target protein, a decrease in the STD signal intensities of the reference ligand is observed. If the IC_50_ value is reached before the equimolar ratio of the reference and the competitor ligand, it can be concluded that the competitor binds more strongly than the selected reference compound to the protein. The strength of the interaction can be quantified by the determination of the dissociation constant value (K_D_).

**Figure 1:**
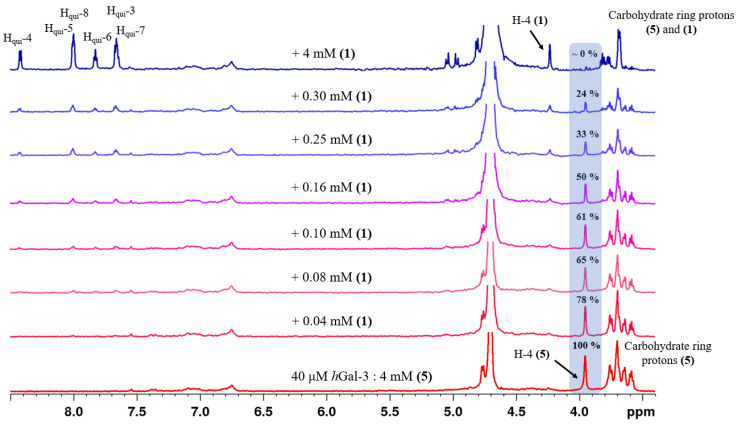
STD ^1^H NMR spectra recorded during the titration experiment of compound **1**. Starting from the bottom, the STD ^1^H NMR spectrum of the sample contained only the target protein (*h*Gal-3) and the reference ligand (**5**) at the concentration of 40 μM and 4 mM, respectively. Moving from the bottom spectrum to the top, the competitor compound (**1**) was used at increasing concentrations as indicated on the figure and displaced the reference ligand (**5**) in the *h*Gal-3 binding site. It resulted in a decrease in the STD NMR signals of the reference ligand (**5**), as illustrated on the H-4 proton signal.

**Figure 2:**
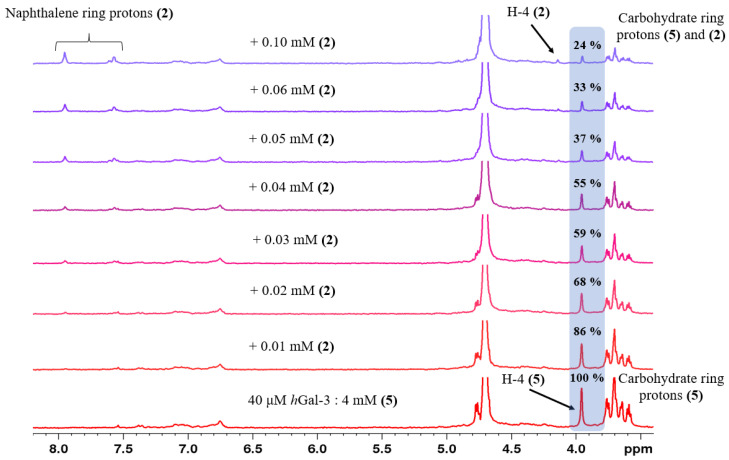
STD ^1^H NMR spectra recorded during the titration experiment of compound **2**. Starting from the bottom, the STD ^1^H NMR spectrum of the sample contained only the target protein (*h*Gal-3) and the reference ligand (**5**) at the concentration of 40 μM and 4 mM, respectively. Moving from the bottom spectrum to the top, the competitor compound (**2**) was used at increasing concentrations as indicated on the figure and displaced the reference ligand (**5**) in the *h*Gal-3 binding site. It resulted in a decrease in the STD NMR signals of the reference ligand (**5**), as illustrated on the H-4 proton signal.

**Figure 3:**
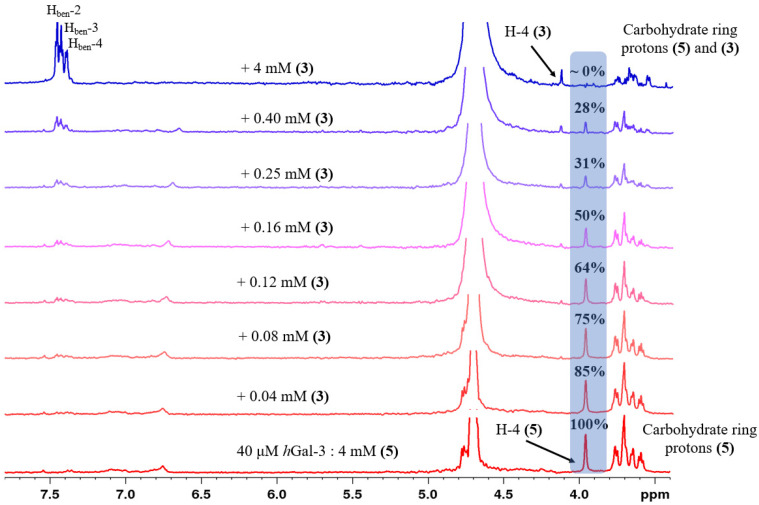
STD ^1^H NMR spectra recorded during the titration experiment of compound **3**. Starting from the bottom, the STD ^1^H NMR spectrum of the sample contained only the target protein (*h*Gal-3) and the reference ligand (**5**) at the concentration of 40 μM and 4 mM, respectively. Moving from the bottom spectrum to the top, the competitor compound (**3**) was used at increasing concentrations as indicated on the figure and displaced the reference ligand (**5**) in the *h*Gal-3 binding site. It resulted in a decrease in the STD NMR signals of the reference ligand (**5**), as illustrated on the H-4 proton signal.

We chose the TDG (**5**), a well-known, moderately bound ligand of *h*Gal-3, as a reference ligand for our competition STD ^1^H NMR experiments. As a first step, we recorded an STD ^1^H NMR spectrum on the starting sample, which always contained 40 μM *h*Gal-3 protein and 4 mM TDG (1:100 protein reference ligand ratio). Then, the titration was started by adding the synthesized competitor ligand into the NMR sample step-by-step, and we performed STD experiments on each solution composition. Figure 1, Figure 2 and Figure 3 show the STD ^1^H NMR spectra recorded in the titration experimental series of compounds **1**–**3**, respectively. The concentration steps of the titration summarized in Table 1 were always fine-tuned to the given competitor ligand to determine the IC_50_ value for each compound as accurately as possible. In each case, the change in the STD signal intensity of the H-4 sugar ring proton of TDG (**5**) was followed because this resonance signal does not overlap in the ^1^H spectra with any other signals. Thus, the 100% STD effect was determined as the H-4 intensity value of TDG (**5**) in the initial sample state, and its relative decrease caused by adding the competitor ligand in increasing concentration was measured in each titration step (Table 1). Unfortunately, we were not able to investigate the fourth aromatic compound (**4**) with the STD NMR method because it was not soluble in water at the appropriate concentration.

**Figure 4:**
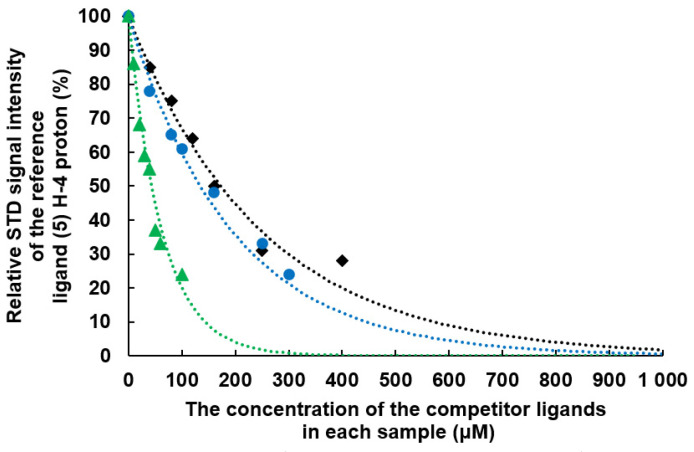
Determination of the IC_50_ values based on the competition between reference ligand (**5)** and compound **1** (blue), **2** (green) and **3** (black) for the binding pocket of *h*Gal-3 of each STD ^1^H NMR experimental series. The IC_50_ values were determined based on the equations of the exponential functions fitted to the points measured. We did not observe any aggregation during the series of experiments of compounds **1** and **3**, so it was possible to completely displace the reference ligand in the binding pocket at 1:1 reference and competition ligand ratios. However, the solubility of compound **2** above 100 µM was uncertain under the given experimental conditions, so the titration experiment was stopped at that point.

By plotting the relative STD ^1^H NMR signal intensities of the H-4 proton of the reference compound (**5**) as a function of the concentration of the given competitor ligand, the IC_50_ values can be determined (Figure 4). The IC_50_ value gives the concentration of the competitor ligand required to halve the signal intensity of the reference ligand in the STD spectrum, so it provides the competitor concentration required for 50% displacement of the reference compound (**5**). IC_50_ values were calculated based on the equation of the exponential function fitted to the points measured.

The dissociation constant of the interaction was calculated according to the following equation (Equation (1)) [40,42]:

(1) $$K_{d(ref.)}=\frac{\left[L_{ref}\right]\times K_{d(comp.)}}{\left({IC}_{50}-K_{d(comp.)}\right)}$$

By rearranging this equation, the K_d_ value of the competitor ligand (K_d(comp.)_) can be obtained (Equation (2)):

(2) $$K_{d(comp.)}=\frac{K_{d(ref.)}\times {IC}_{50}}{\left(\left[L_{ref}\right]+K_{d(ref.)}\right)}$$

where K_d(ref.)_ refers to the dissociation constant of the reference ligand, L_ref_ is the concentration of the reference ligand, K_d(comp.)_ is the dissociation constant of the competitor compound, and IC_50_ is the concentration of the competitor compound at half-signal intensity.

The calculated IC_50_ and K_D_ values of the competitor ligands investigated, and their relative *h*Gal-3 binding affinities referenced to TDG, are summarized in Table 2. The results show that the synthesized aromatic TDG derivatives (compounds **1**, **2** and **3**) bind more strongly to the *h*Gal-3 protein than the reference TDG. Therefore, it is proven herein that an aromatic functional group at the 3,3′ positions of TDG positively influences the strength of the ligand–protein interaction, in line with previous findings [11,37]. The strongest binding affinity has been determined for the (naphthalen-2-yl)methyl derivative (**2**), which showed 94 times stronger binding than TDG (**5**). However, the 30- and 24-fold gain of interaction strength of the (quinolin-2-yl)methyl (**1**) and benzyl (**3**) derivative, respectively, compared to the reference ligand (**5**) is also remarkable.

### 2.3. Molecular Docking Simulations

In order to rationalize the obtained binding strengths of the investigated compounds, we performed molecular docking calculations and analyzed the obtained docking poses in terms of protein–ligand interactions. By using the binding site of the complex structure of TDG and *h*Gal-3 (4JC1) in the docking calculations, we implicitly assumed that the canonical binding mode of TDG is preserved for all investigated compounds. The goal of the docking calculations was to rationalize how the aromatic substituents influence the otherwise conserved binding and how the differences in the binding strength of the compounds could be explained.

**Figure 5:**
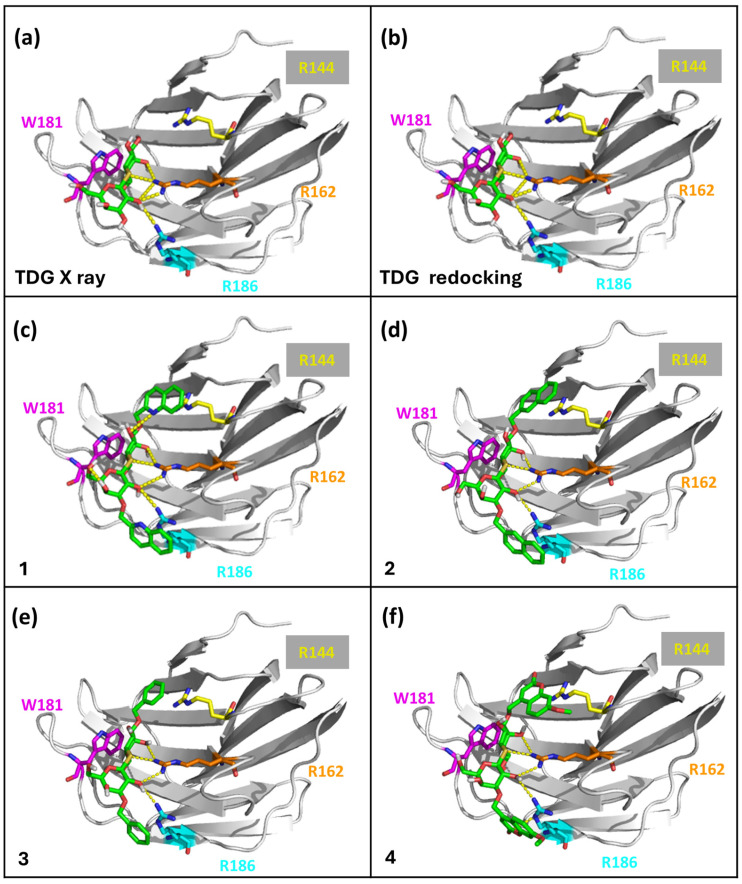
Comparison of (**a**) the binding mode of TDG as observed in the X-ray crystallographic structure with (**b**) the redocking of TDG and the best docking poses of the derivatives **1** in (**c**), **2** in (**d**), **3** in (**e**) and **4** in (**f**). The following key interactions are observed: a stacking interaction between the sidechain of W181 (in magenta) and the inner galactose residue, canonical hydrogen bonding interactions with R162 (in orange) and cation-π interactions formed between the aromatic sidechains and arginine residues R144 (in yellow) and R186 (in cyan). The aromatic groups are in optimal position to interact with the guanidium groups of the arginine residues due to the size and geometry of methylene linker groups.

To test the accuracy of our docking workflow, we performed redocking calculations to reproduce the binding mode of TDG to the Carbohydrate Recognition Domain (CRD) of *h*Gal-3. The RMSD between the TDG atoms of the crystal structure (4JC1) and the redocked pose was 0.70 A. We used the positions of the rings and that of the linker atoms of TDG as observed in the crystal structure as a reference to evaluate the docking poses of the investigated compounds. The observed canonical interactions of TDG (Figure 5a) include a stacking interaction between the TRP-181 sidechain and the apolar side of the sugar ring [44]. Another conserved interaction present for TDG is a hydrogen bond between ARG-162 and the ligand (Figure 5a). The hydrogen donor in the interaction is the guanidium group, and the acceptors are the 4-OH and pyranose oxygen on the proximal galactose ring and the 2-OH on the distal galactose ring. Both interactions are reproduced well in the redocking pose of TDG (Figure 5b), as well as for all of the synthesized derivatives (Figure 5c–f).

**Figure 6:**
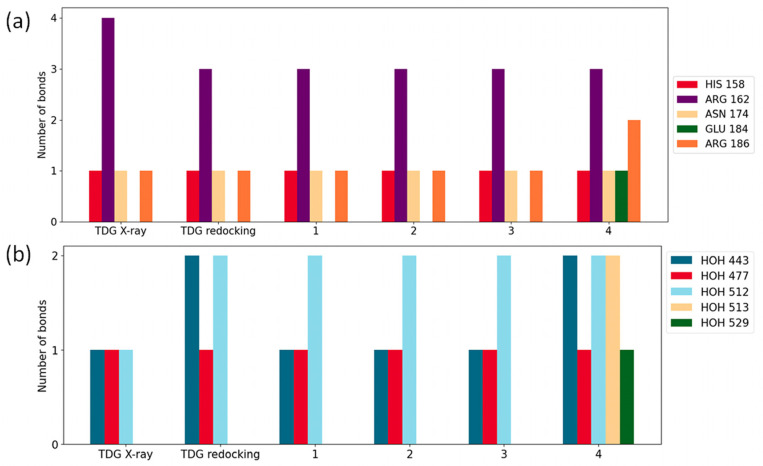
Number of hydrogen bonds detected. (**a**) The number of hydrogen bonds between the ligands and the protein. (**b**) The number of hydrogen bonds between the ligands and the water molecules.

Additional hydrogen bonds are observed in multiple protein sidechains and water molecules, as summarized in Figure 6. The interacting residues are HIS-158, ARG-162, ASN-174, GLU-184, ARG-186 and the water bridges to HOH 443, HOH 477, HOH 512, HOH 513 and HOH 529. Due to the larger size of the (7-methoxy-2H-1-benzopyran-2-on-4-yl)methyl group, in the case of compound **4**, the interactions can be observed with more distant protein residues, such as GLU-184, and with a larger number of water molecules.

Furthermore, the aromatic moieties in the studied derivatives are able to form a cation–π interaction [45,46] with the cationic sidechains of the arginine residues in the binding pocket (Figure 5). The two interacting residues are ARG-144 and ARG-186, which are optimally positioned in the binding site with the symmetrical aromatic substituents for all four compounds (**1–4**). These additional cation–π interactions likely explain the higher binding strengths observed experimentally by NMR for the synthesized derivatives with aromatic substituents compared to TDG. A deeper examination of the potential correlation between the determined K_d_ values and the distances between aromatic substituents and cationic sidechains highlights a significant relationship. Notably, this correlation emphasizes the crucial role of cation–π interactions in the aromatic ligand affinity. Larger polycyclic aromatic groups offer a more extensive surface area for interaction, resulting in stronger binding energies. In line with this, the benzyl moiety of compound **3**, which has a comparatively smaller interaction surface, exhibits a lower affinity to *h*Gal-3 than the (naphthalene-2-yl)methyl group. The nitrogen atom within the quinoline moiety of compound **1** disrupts the homogenous charge density of the aromatic group by attracting the electron density to itself. This effect leads to an electron redistribution, resulting in a less favorable interaction energy compared to the naphthalene ring.

**Figure 7:**
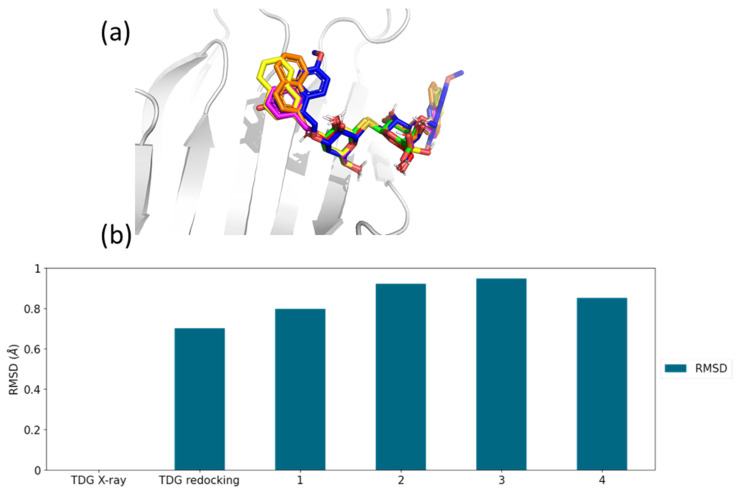
Overlay (**a**) and RMSD values (**b**) of the crystal structure of TDG and the docking poses of the derivatives synthesized. As the top diagram shows, the thiodigalactoside scaffold of all ligands is located in the same binding pocket and preserves the canonical binding mode as supported by the RMSD values, which are below 1 Å for all ligands.

The overlay of the best docking poses for the four aromatic derivatives indicates that the canonical binding mode of TDG is preserved, as illustrated in Figure 7a. The RMSD of the common core scaffold atoms between the crystal structure of TDG and the best docking poses of the aromatic derivatives studied is below 1 Å (Figure 7b).

The scores calculated for the docking poses, the corresponding dissociation constants and the experimentally determined dissociation constants are shown in Table 3. The docking scores were treated as crude approximations of the binding free energies and were converted to dissociation constants at 303 K, the temperature used in the NMR experiments. The calculated and the experimentally determined dissociation constants are in fair agreement. The docking score somewhat underestimates the binding strength of the redocked pose of TDG and overestimates the binding calculated for the pose found in the X-ray structure. This shows that the docking score is sensitive to slight changes in the binding mode of the core sugar scaffold. The obtained scores also highlight the impact of the cation–π interactions on the binding free energy. It is noteworthy that ligands containing two fused aromatic rings, ligands **1**, **2** and **4**, are predicted to have an order-of-magnitude higher binding free energy than ligand **3** with a benzyl substituent, probably due to larger surfaces available for interactions. Furthermore, ligands **1** and **2**, assessed through the molecular mechanics-based docking function, exhibit close binding free energy values and comparable dissociation constants for similarly sized aromatic substituents. However, the order of their dissociation constants contradicts expectations based on electron densities, likely due to the molecular mechanics approach and the ligands not having calculated electron densities.

## 3. Materials and Methods

### 3.1. Chemical Synthesis

#### 3.1.1. Bis-(2,3,4,6-Tetra-O-Acetyl-β-D-Galactopyranosyl)-Disulfide (**8**)

2,3,4,6-Tetra-*O*-acetyl-β-D-galactopyranosyl isothiouronium bromide (**6**) (8.0 g, 11.24 mmol) was suspended in CH_2_Cl_2_ (30 mL) and added to a solution of Na_2_S_2_O_5_ (20 g, 105.26 mmol) inH_2_O (63 mL). The reaction mixture was stirred at reflux temperature for 3 h. After consumption of the starting material, the reaction mixture was diluted with CH_2_Cl_2_ (20 mL), and the organic phase was washed with water (2 × 40 mL), dried over MgSO_4_, filtered and evaporated to yield thiol **7** (4.74 g, 87%) as a colorless oil. The crude thiol **7** (4.74 g, 13.02 mmol) was dissolved in EtOAc (43 mL), and NaI (0.019 g, 0.13 mmol) and 33% H_2_O_2_ (1.25 mL) were added. The reaction mixture was stirred for 1 h at room temperature. The solution was treated with saturated Na_2_S_2_O_5_ solution; EtOAc (20 mL) was added; and the organic phase was washed with H_2_O (3 × 35 mL), dried over MgSO_4_, filtered and evaporated. Compound **8** [36] (4.40 g, 93%) was isolated as white foam. [α]^24^_D_ -63.9 (c 0.20, CHCl_3_). R_f_ 0.51 (*n-*hexane: EtOAc 1:1)

#### 3.1.2. Bis-(2,3,4,6-Tetra-O-Acetyl-β-D-Galactopyranosyl)-Sulfane (**9**)

The chemical was prepared in gram scale according to a modified literature procedure, using galactosyl bromide instead of galactosyl iodide [4]. Bis-(2,3,4,6-tetra-*O*-acetyl-β-D-galactopyranosyl)-disulfide (**8**) (4.05 g, 5.58 mmol) was dissolved in dry acetonitrile (120 mL) under argon. After 10 min, NaBH_4_ (0.67 g, 17.12 mmol) was added to the mixture and stirred for 30 min. 1-Bromo-2,3,4,6-tetra-*O*-acetyl-α-D-galactopyranose (**10**) (4.08 g, 9.93 mmol) was added to the mixture and stirred for a further 30 min. Another portion of NaBH_4_ (0.67 g, 17.12 mmol) was added to the reaction mixture and stirred overnight at room temperature. When TLC showed complete conversion of the starting materials, it was treated with 96% acetic acid and evaporated. The residue was dissolved in CH_2_Cl_2_ (150 mL), washed with water (2 × 100 mL), dried over MgSO_4_, filtered and evaporated. The crude product was purified by crystallization from dry CH_3_OH to yield compound **9** (3.36 g, 87%) as a white powder. R_f_ 0.26 (*n-*hexane: EtOAc 1:1); m.p. 195–197 °C, lit. [4] m.p. 196–197 °C; [α]^24^_D_ -7.5 (c 0.20, CHCl_3_); lit. [4] [α]^20^_D_ -14.0 (c 0.65, CHCl_3_).

ESI-HRMS *m*/*z* [M+Na]^+^ calc. for (C_28_H_38_O_18_SNa) 717.1677, found 717.1675.

MALDI HRMS found 717.1678 [35].

#### 3.1.3. Bis-(β-D-Galactopyranosyl)-Sulfane (**5**, Thiodigalactoside, TDG)

To a stirred solution of **9** (3 g, 4.32 mmol), MeONa (pH~9) was added in dry MeOH (25 mL) and stirred for 2 h at room temperature. The reaction mixture was neutralized with Amberlyst^®^ 15 H^+^ ion-exchange resin, filtered and evaporated; the crude product was purified by crystallization from dry CH_3_OH to yield **5** (TDG, 1.41 g, 91%) as a white powder. R_f_ 0.25 (EtOAc:MeOH: H_2_O 15:5:1.6); [α]^24^_D_ -30.5 (c 0.20, DMSO);

^1^H-NMR (700 MHz, D_2_O, 298 K): *δ* 4.74 (d, *J* = 9.9 Hz, 2H, H-1); 3.92 (d, *J* = 3.3 Hz, 2H, H-4); 3.72 (m, 2H, H-6a); 3.69–3.63 (overlapped signals, 4H, H-5, H-6b); 3.61 (dd, *J* = 9.6 Hz, *J* = 3.3 Hz, 2H, H-3); 3.53 (t, *J* = 9.7 Hz, 2H, H-2); ^13^C-NMR (175 MHz, D_2_O, 298 K): δ 83.6 (C-1); 79.1 (C-5); 73.9 (C-3); 69.7 (C-2); 68.9 (C-4); 61.3 (C-6). ESI-HRMS *m*/*z* [M+Na]^+^ calc. for (C_12_H_22_O_10_SNa) 381.0831, found 381.0834. MALDI-HRMS *m*/*z* [M+Na]^+^ calc. for (C_12_H_22_O_10_SNa) 381.0831 [35], found 381.0835.

#### 3.1.4. General Procedure for the Synthesis of 3,3′-di-O-Aralkyl-Thiodigalactosides **1**–**4** [11,14]

3,3′-di-*O*-aralkyl-thiodigalactosides (**1**–**4**) were synthesized under optimized reaction conditions.

Compound **5** (TDG) (1 equiv., 3.00 g, 8.37 mmol) was dissolved in dry methanol (270 mL), and dibutyltin(IV) oxide (3 equiv., 6.27 g, 25.19 mmol) was added, then stirred at reflux temperature for 3 h. After removing the solvent, the acetal was further reacted with the corresponding arylmethylation reagent using the optimized conditions: it was dissolved in dry 1,4-dioxane, and arylmethyl halide reagent (3 equiv.) and TBAB (0.75 equiv.) were added in an argon atmosphere and stirred at 85 °C. After 8 h, the reaction mixture was evaporated and the residues dissolved in ethyl acetate (50 mL), washed with distilled water (2 × 15 mL), dried over MgSO_4_, filtered and evaporated. The crude products were purified by flash column chromatography (Merck, Darmstadt, Germany) (CH_2_Cl_2_:MeOH 9:1) to yield compounds **1**–**4**.

#### 3.1.5. Bis-{3-O-[(Quinoline-2-yl)Methyl]-β-D-Galactopyranosyl}-Sulfane (**1**)

TDG (**5**, 0.21 g, 0.59 mmol) was reacted according to the general procedure, using 0.42 g (1.69 mmol) of dibutyltin(IV) oxide in 10 mL of dry methanol for the tin-acetylation step, then 0.39 g (1.77 mmol) of 2-(bromomethyl)quinoline and 0.49 g (1.53 mmol) of TBAB in 10 mL of dry 1,4-dioxane for the arylmethylation step. Compound **1** (0.24 g, 63%) was isolated as brownish powder. [α]^24^_D_ + 8.76 (c 0.10, DMSO); R_f_ 0.85 (CH_2_Cl_2_:MeOH 9:1).

^1^H-NMR (700 MHz, DMSO, 298 K): *δ* 8.45 (d, *J* = 8.4 Hz, 2H, H_qui_-4); 8.08–8.01 (overlapped signals, 4H, H_qui_-5, H_qui_-8); 7.86 (t, *J* = 6.8 Hz, 2H, H_qui_-6); 7.72 (d, *J* = 8.6 Hz, 2H, H_qui_-3); 7.69 (t, *J* = 7.7 Hz, 2H, H_qui_-7); 5.09–4.94 (dd, *J* = 13.9 Hz, 4H, CH_2A,B_); 4.82 (d, *J* = 10.0 Hz, 2H, H-1); 4.24 (d, *J* = 2.8 Hz, 2H, H-4); 3.83 (t, *J* = 9.5 Hz, 2H, H-2); 3.78 (m, 2H, H-6a), 3.73–3.64 (overlapped signals, 6H, H-6b, H-5, H-3); ^13^C-NMR (175 MHz, DMSO, 298 K): δ 160.0 (C_qui_-2); 147.5 (C_qui_-4a); 139.7 (C_qui_-4); 132.2 (C_qui_-6); 129.7 (C_qui_-5); 129.0 (C_qui_-8a); 128.6 (C_qui_-7); 128.5 (C_qui_-8); 121.7 (C_qui_-3); 84.6 (C-1); 84.2 (C-3); 80.3 (C-5); 72.5 (CH_2_); 70.3 (C-2); 66.9 (C-4); 62.6 (C-6). 

ESI-HRMS: *m*/*z* [M+Na]^+^ calc. for (C_32_H_36_N_2_O_10_SNa) 663.1988, found 663.1980.

#### 3.1.6. Bis-{3-O-[(Naphtalene-2-yl)Methyl]-β-D-Galactopyranosyl}-Sulfane (**2**)

TDG (**5**, 0.40 g, 1.11 mmol) was reacted according to the general procedure, using 0.84 g (3.35 mmol) of dibutyltin(IV) oxide in 20 mL of dry methanol for the tin-acetylation, then 0.74 g (3.34 mmol) of 2-(bromomethyl)naphthalene and 0.98 g (3.06 mmol) of TBAB in 20 mL of dry 1,4-dioxane for the arylmethylation. Compound **2** (0.48 g, 60%) was isolated as light brownish syrup. [α]^24^_D_ + 2.65 (c 0.18, DMSO); lit. [11] [α]^20^_D_ + 2.60 (c 0.23, MeOH); R_f_ 0.76 (CH_2_Cl_2_:MeOH 9:1).

^1^H-NMR (700 MHz, DMSO, 303 K): *δ* 8.45 (d, *J* < 1 Hz, 2H, H_napht_-1); 7.92–7.86 (overlapped signals, 6H, H_napht_-4, H_napht_-5, H_napht_-8); 7.60 (dd, *J* = 8.4 Hz, *J* = 1.2 Hz, 2H, H_napht-_3); 7.54–7.47 (overlapped signals, 4H, H_napht_-6, H_napht_-7); 5.17 (d, 2H, OH); 4.90–4.74 (dd, *J* = 13.6 Hz, 4H, CH_2A,B_), 4.64–4.55 (overlapping signals, 6H, H-1, H-3, OH); 4.03 (d, *J* = 2.8 Hz, 2H, H-4), 3.33 (m, 2H, H-5); 3.58–3.47 (overlapping signals, 6H, H-6a, H-6b, OH); ^13^C-NMR (175 MHz, DMSO, 303 K): δ 137.2 (C_napht_-2); 133.3 (C_napht_-8a); 132.9 (C_napht_-4a); 128.1; 128.0 (C_napht_-8, C_napht_-5, C_napht_-4); 126.5 (C_napht_-6, C_napht_-3); 126.2 (C_napht_-7, C_napht_-1); 83.4 (C-1); 83.0 (C-3); 79.6 (C-5); 70.9 (CH_2_); 69.8 (C-2); 65.5 (C-4); 60.7 (C-6). 

ESI-HRMS: *m*/*z* [M+Na]^+^ calc. for (C_34_H_38_O_10_SNa) 661.2083, found 661.2076.

#### 3.1.7. Bis-(3-O-Benzyl-β-D-Galactopyranosyl)-Sulfane (**3**)

TDG (**5**, 1.14 g, 3.18 mmol) was reacted according to the general procedure, using 2.39 g (9.62 mmol) of dibutyltin(IV) oxide in 60 mL of dry methanol for the tin-acetylation, then benzylbromide (1.13 mL, 3 equiv.) and 0.77 g (2.39 mmol) of TBAB in 60 mL of dry 1,4-dioxane for the arylmethylation. Compound **3** (0.51 g, 30%) was isolated as yellowish syrup. [α]^24^_D_ -0.65 (c 0.13, DMSO); lit. [11] [α]^20^_D_ -0.67 (c 0.22, MeOH); R_f_ 0.52 (CH_2_Cl_2_:MeOH 9:2).

^1^H-NMR (700 MHz, D_2_O, 298 K): *δ* 7.40 (d, *J* = 7.3 Hz, 2H, H_ben_-2); 7.37 (t, *J* = 7.2 Hz, 2H, H_ben_-3); 7.33 (t, *J* = 7.3 Hz, 2H, H_ben_-4); 4.72–4.58 (overlapped signals, 6H, H-1, CH_2A,B_); 4.07 (d, *J* = 3.1 Hz, 2H, H-4); 3.69 (dd, 2H, H-6a, *J* = 11.7 Hz, *J* = 8.1 Hz); 3.64–3.56 (overlapped signals, 6H, H-6b, H-5, H-2); 3.49 (dd, *J* = 9.4 Hz, *J* = 3.1 Hz, 2H, H-3); ^13^C-NMR (175 MHz, D_2_O, 298 K): δ 137.3 (C_ben_-1); 128.7 (C_ben_-4); 128.6 (C_ben_-3); 128.3 (C_ben_-2); 83.5 (C-1); 81.0 (C-3); 79.0 (C-5); 71.2 (CH_2_); 68.8 (C-2); 65.7 (C-4); 61.3 (C-6). 

ESI-HRMS: *m*/*z* [M+Na]^+^ calc. for (C_26_H_34_O_10_Na) 561.1770, found 561.1780.

#### 3.1.8. Bis-{3-O-[(7-Methoxy-2H-1-Benzopyran-2-on-4-yl)Methyl]-β-D-Galactopyranosyl}-Sulfane (**4**) [37]

TDG (**5**, 0.50 g, 1.40 mmol) was reacted according to the general procedure, using 1.04 g (4.19 mmol) of dibutyltin(IV) oxide in 25 mL of dry methanol for the tin-acetylation, then 1.13 g (4.19 mmol) of 4-bromomethyl-7-methoxycoumarin and 1.22 g (3.80 mmol) of TBAB in 25 mL of dry 1,4-dioxane for the arylmethylation. Compound **4** (0.48 g, 47%) was isolated as yellowish powder. R_f_ 0.45 (CH_2_Cl_2_:MeOH 9:1).

^1^H-NMR (500 MHz, DMSO, 298 K): *δ* 7.67 (d, *J* = 8.7 Hz, 2H, H_cum_-5); 7.02 (s, 2H, H_cum_-8); 6.95 (d, *J* = 8.5 Hz, 2H, H_cum_-6); 6.64 (s, 2H, H_cum_-3); 5.37 (d, OH-2, 2H); 5.00–4.79 (dd, *J* 15.9 Hz, 4H, CH_2A,B_); 4.74 (d, 2H, OH-4,); 4.76 (t, 2H, OH-6); 4.62 (d, *J* = 9.8 Hz, 2H, H-1); 4.09 (d, *J* 3.0 Hz, 2H, H-4); 3.82 (s, 6H, OCH_3_); 3.66 (m, 2H, H-2); 3.60–3.42 (overlapping signals, 4H, H-6a, H-6b); 3.43 (m, 2H, H-3). ^13^C-NMR (125 MHz, DMSO, 298 K): δ 162.2 (C_coum_-2); 160.5 (C_coum_-7); 154.9 (C_coum_-4); 153.3 (C_coum_-8a); 125.6 (C_coum_-5); 112.2 (C_coum_-6); 110.7 (C_coum_-3); 108.9 (C_coum_-8); 100.8 (C_coum_-4a); 83.3 (C-3); 82.8 (C-1); 79.0 (C-5); 69.1 (C-2); 66.1 (CH_2_); 64.9 (C-4); 60.3 (C-6); 55.9 (OCH_3_). 

ESI-HRMS: *m*/*z* [M+Na]^+^ calc. for (C_34_H_38_O_16_SNa) 757.1778, found 757.1780. Found 757.1781 [37].

### 3.2. General Methods

Optical rotation was measured at room temperature with a Perkin-Elmer 241 automatic polarimeter. TLC analysis was performed on Kieselgel 60 F254 (Merck) silica gel plates with visualization by immersing in a sulfuric acid solution (5% in EtOH), followed by heating. Column chromatography was performed on silica gel 60 (Merck 0.063–0.200 mm). Organic solution was dried over MgSO_4_ and concentrated under reduced pressure. 1D ^1^H, *J*-modulated ^13^C, 2D ^1^H-^1^H COSY, ^1^H-^13^C HSQC, ^1^H-^13^C HSQC-CLIP-COSY (Clean In-Phase Correlation Spectroscopy) [47], ^1^H-^13^C HMBC NMR spectra were recorded with Bruker Avance Neo 700 MHz and Bruker Avance II 500 MHz spectrometers (Bruker, Billerica, MA, USA). Chemical shifts are referenced to Me_4_Si or DSS (0.00 ppm for ^1^H) and to solvent signals (DMSO: 49.51 ppm for ^13^C). The 1D and 2D NMR spectra of the synthesized compounds can be found in the Supplementary Materials (Figures S1–S5). ESI-QTOF MS measurement was carried out on a maXis II UHR ESI-QTOF MS instrument (Bruker, Billerica, MA, USA) in positive ionization mode.

### 3.3. ^1^H STD STD NMR Experiments

All ^1^H STD NMR experiments were recorded on a Bruker Avance Neo 700 MHz spectrometer equipped with a 5 mm z-gradient (TCI) triple-tuned Prodigy cryoprobe. The measurements were performed at a temperature of 303 K. The data acquisition and processing were performed with TopSpin 3.6.2 and 4.1.1 software. Samples were prepared in 10 mM phosphate buffer solution (140 mM NaCl/KCl, pH = 7.4) in D_2_O. The initial sample contained 40 μM of *h*Gal-3 protein and 4 mM of compound **5** for each experiment. Competitor ligands were added to the initial samples from stock solutions of different concentrations so that the smallest volume (2 μL) could be added in each titration step. The exact concentration of the competitor ligands in the solution measured can be found in the respective figures. The protein resonances were selectively irradiated in competition STD experiments by Eburp (90°) excitation pulses, with a length of 50 ms each with a maximum B_1_ field strength of 75 Hz yielding a total irradiation time of 3 s. The off-resonance pulse frequency was set to −40 ppm and the on-resonance frequency to 0 ppm. Off- and on-resonance data were recorded at alternate scans, and the corresponding FIDs were collected in separate memories of subsequent processing files, and STD spectra were produced after subtraction of the two subspectra. Competition STD spectra were typically recorded with 1200 repetitions (NS = 8, L4 = 150) to ensure an adequate signal-to-noise ratio for the analysis.

### 3.4. Docking Calculations

We used the X-ray crystallographic structures of TDG bound to h-Gal3 [48] as a basis for molecular dockings. Crystallographic water molecules were kept for the calculations. The molecules were modified using GaussView 6 [49]. We performed dockings in the Python version of Autodock Vina [50,51] with the Vina scoring function. We set the simulation box as a cube of size 30 Å, with a center defined as the geometric center of the TDG ligand with a grid spacing of 0.1 Å. We performed two types of dockings: an explorative docking with exhaustiveness of 8, number of poses of 1000 and a minimum RMSD of 0.005, and a refined docking with exhaustiveness of 32, number of poses of 100 and a minimum RMSD of 0.1. The maximum number of evaluations were set to 0 with the seed at “1”, and the energy range for saving the poses was 20.0 kcal/mol. Minimization of the obtained docking poses was carried out with the same simulation box as the dockings. Poses were selected both from the explorative and the refined docking output in two steps using multiple selection criteria. In the first step, three criteria were used to select 5 poses. The first selection was carried out based on the RMSD between the common atoms of a reference structure and the derivative, then the lowest distance between the sugar ring and the TRP-181 indole ring, and finally, the most favorable docking energy was used. In the second step, the best pose among these 5 was selected according to the highest number of interactions between the ligand and protein, using the proximal galactose moiety of the X-ray structure of the TDG as a reference. The full docking workflow was as follows: we created the starting molecules, minimized them, then performed an explorative docking, followed by a pose selection with previously determined selection rules, using 7 Å as the RMSD cutoff. We performed a fine docking from the best pose using another round of pose selection, and minimization. All minimized best poses were used for extraction of docking scores and the number of protein–ligand interactions. Pose scores were determined by the scoring function of Autodock Vina with the Vina scoring function [50,51]. We used an in-house written code for the determination of protein–ligand interactions, including hydrogen bonds, cation–π interactions, apolar interactions and stacking interactions.

## 4. Conclusions

Here, we report the synthesis of a novel 3,3′-di-*O*-(*N*-heterocyclic)-thiodigalactoside derivative, bis-{3-*O*-[(quinolin-2-yl)methyl]-β-D-galactopyranosyl}-sulfane (**1**), under optimized reaction conditions, starting from thiodigalactoside (TDG, **5**) via stannylidene acetal with three equivalent 2-(bromomethyl)quinoline. Three additional aromatic TDG derivatives (**2**, **3** and **4**) were also produced for affinity and structural studies in moderate-to-good yields under these optimized reaction conditions. We have also developed a simple, inexpensive synthetic route for the large-scale preparation of TDG (**5**). Starting from bis-(2,3,4,6-tetra-*O*-acetyl-β-D-galactopyranosyl)-disulfide and 1-bromo-2,3,4,6-tetra-*O*-acetyl-α-D-galactopyranose, acetyl-protected TDG was obtained in good yield, then acetyl protective group removal according to the Zemplén method afforded TDG in excellent yield.

Competition STD ^1^H NMR experiments were performed to determine the affinities of the synthesized aralkyl carbohydrate derivatives to *h*Gal-3 protein. The strongest affinity was measured in the case of compound **2**, which showed 94 times (Kd = 0.55 μM) stronger binding compared to the reference TDG (**5**) (Kd = 51.4 μM) [43]. The 30- (Kd = 1.70 μM) and 24-fold (Kd = 2.19 μM) increase in the interaction strength compared to the reference ligand (**5**) for compound **1** and compound **3**, respectively, is also an interesting result. Thus, we have proved that aralkyl substitution in the 3 and 3′ positions of TDG provided such carbohydrate derivatives that had a significantly stronger binding affinity to *h*Gal-3 than the basic TDG (**5**), in line with previous findings. However, the introduction of the quinoline ring as a substituent has not improved the binding affinity to *h*Gal-3. Contrary to our plans, the benzopyranon-substituted TDG derivative (**4**) could not be investigated by the STD NMR method due to its low water solubility.

Molecular docking simulations demonstrated that all thiodigalactoside derivatives had similar spatial orientations and interactions with *h*Gal-3 in the binding pocket. Our findings highlight the crucial role of cation–π interactions in the ligand binding of aralkyl thiodigalactoside derivatives and offer a molecular-level understanding of the varying affinities observed among different ligands. This observation could be a decisive feature in the subsequent development of highly efficient inhibitor molecules of *h*Gal-3. Therefore, in the future, we plan to synthesize and investigate such TDG derivatives, in which the aromatic parts possess higher aromatic character then quinoline.

## Figures and Tables

**Table 1:** Relative intensity changes of the H-4 proton’s ^1^H STD NMR signal of the reference compound (**5**), TDG, upon titration with the competitor ligands (**1**–**3**). The concentration of *h*Gal-3 and TDG (**5**) were 40 μM and 4 mM, respectively, in each experimental series.

Concentration of 1 in the Sample [μM]	Relative STD Intensity of H-4 of TDG Titrated by 1 [%]	Concentration of 2 in the Sample [μM]	Relative STD Intensity of H-4 of TDG Titrated by 2 [%]	Concentration of 3 in the Sample [μM]	Relative STD Intensity of H-4 of TDG Titrated by 3 [%]
0	100	0	100	0	100
40	78	10	86	40	85
80	65	20	68	80	75
100	61	30	59	120	64
160	48	40	55	160	50
250	33	50	37	250	31
300	24	60	33	400	28
4000	0	100	24	4000	0

**Table 2:** Comparison of K_d_ values of each ligand (included the reference ligand **(5)**).

Inhibitor Ligand	IC_50_ [μM]	K_d_ [μM]	Relative Affinity ^2^	Valency ^3^
Compound (**5**) ^1^	-	51.4 [43]	1	2
Compound (**1**)	134	1.70	30	2
Compound (**2**)	43	0.55	94	2
Compound (**3**)	172	2.19	24	2

^1^ Reference ligand, ^2^ K_d_ (ref.)/K_d_ (comp.), ^3^ Number of galactose units per molecules.

**Table 3:** Score values from docking and comparison of K_d_ values calculated from the docking scores and determined from NMR (compounds **1**–**3**) and ITC (TDG) [43] measurements.

Inhibitor Ligand	Score [Kcal/mol]	K_d-score_ [μM]	K_d_ [μM]
**1**	−7.548	3.60	1.70
**2**	−7.303	5.40	0.55
**3**	−6.556	18.68	2.19
**4**	−7.700	2.79	n.a.
**TDG-redocking**	−6.116	38.79	51.40
**TDG X-ray**	−5.788	66.88	51.40

## Data Availability

All data can be directly obtained by contacting the authors.

## Supplementary Materials

The following supporting information can be downloaded at: https://www.mdpi.com/article/10.3390/ijms25031742/s1.
