# Supplementary material for: Saturation Transfer Difference NMR and Molecular Docking Interaction Study of Aralkyl-Thiodigalactosides as Potential Inhibitors of the Human-Galectin-3 Protein
Source: Int J Mol Sci. 2024 Feb 1;25(3):1742. doi: 10.3390/ijms25031742 (PMC10855533; doi:10.3390/ijms25031742)
Supplement: Supplementary file 1 [file ijms-25-01742-s001.zip › ijms-2828712-supplementary.pdf]

Table of Content

1D and 2D NMR spectra of synthesised compounds (1) – (5). 2

Figure S1. ^{1}H (a), ^{1}H-^{13}C HSQC (b), ^{1}H-^{13}C HMBC (c), ^{1}H-^{13}C HSQC-CLIP-COSY (d) NMR spectra of (**5**) 2

Figure S2. ^{1}H (a), ^{13}C (b), ^{1}H-^{1}H COSY (c), ^{1}H-^{13}C HSQC (d), ^{1}H-^{13}C HMBC (e), ^{1}H-^{13}C HSQC-CLIP-COSY (f) NMR spectra of (**1**) 3

Figure S3. ^{1}H (a), ^{13}C (b), ^{1}H-^{13}C HSQC (c) ^{1}H-^{13}C HMBC (d), ^{1}H-^{13}C HSQC-CLIP-COSY (e) NMR spectra of (**2**) 6

Figure S4. ^{1}H (a), ^{13}C (b), ^{1}H-^{13}C HSQC (c) ^{1}H-^{13}C HMBC (d), ^{1}H-^{13}C HSQC-CLIP-COSY (e) spectra of (**3**) 8

Figure S5. ^{1}H (a), ^{13}C (b), ^{1}H-^{1}H COSY (c), ^{1}H-^{13}C HSQC (d) NMR spectra of (**4**) 10

1D and 2D NMR spectra of synthesised compounds (1) – (5).

Figure S1. ^{1}H (a), ^{1}H-^{13}C HSQC (b), ^{1}H-^{13}C HMBC (c), ^{1}H-^{13}C HSQC-CLIP-COSY (d) NMR spectra of (**5**)

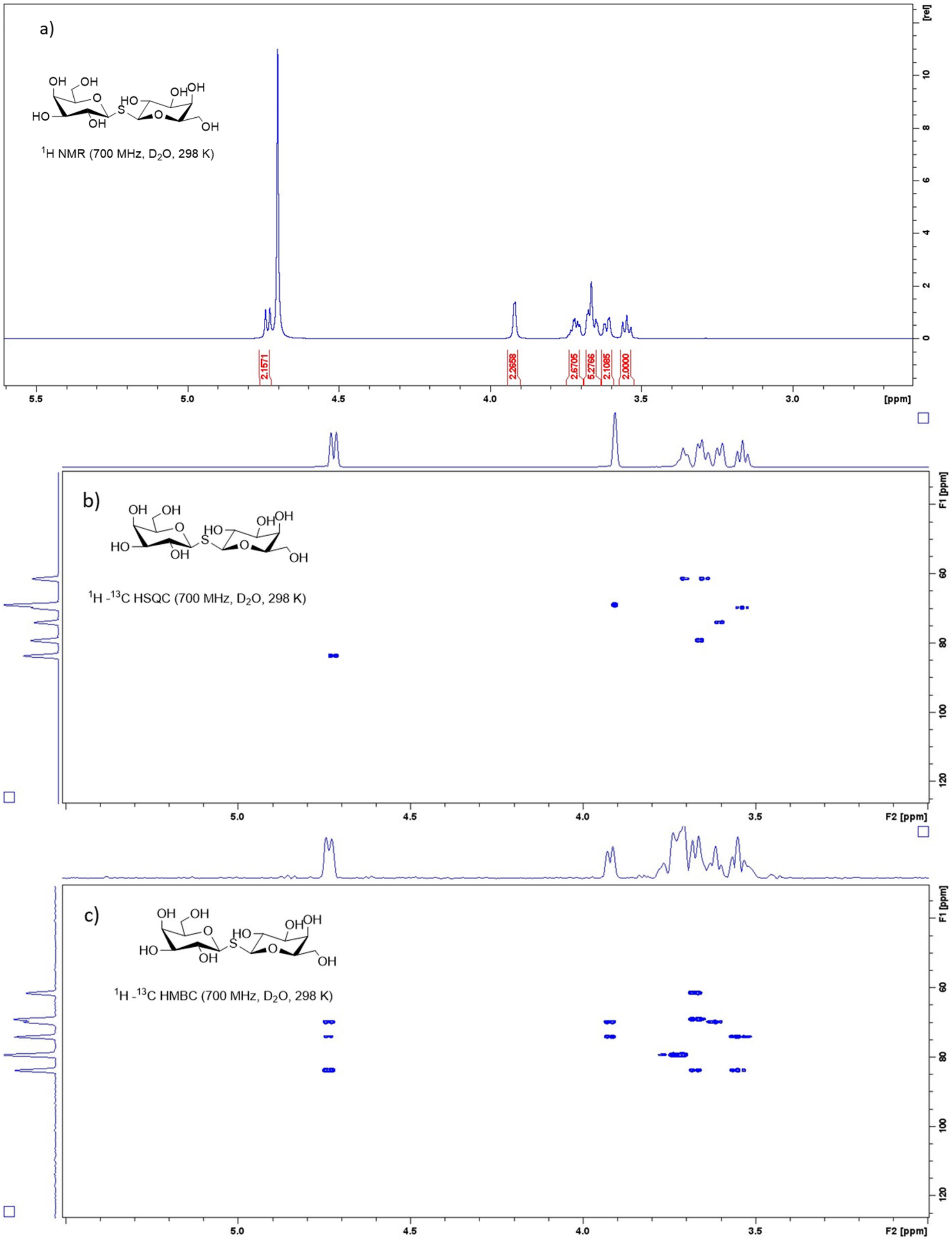

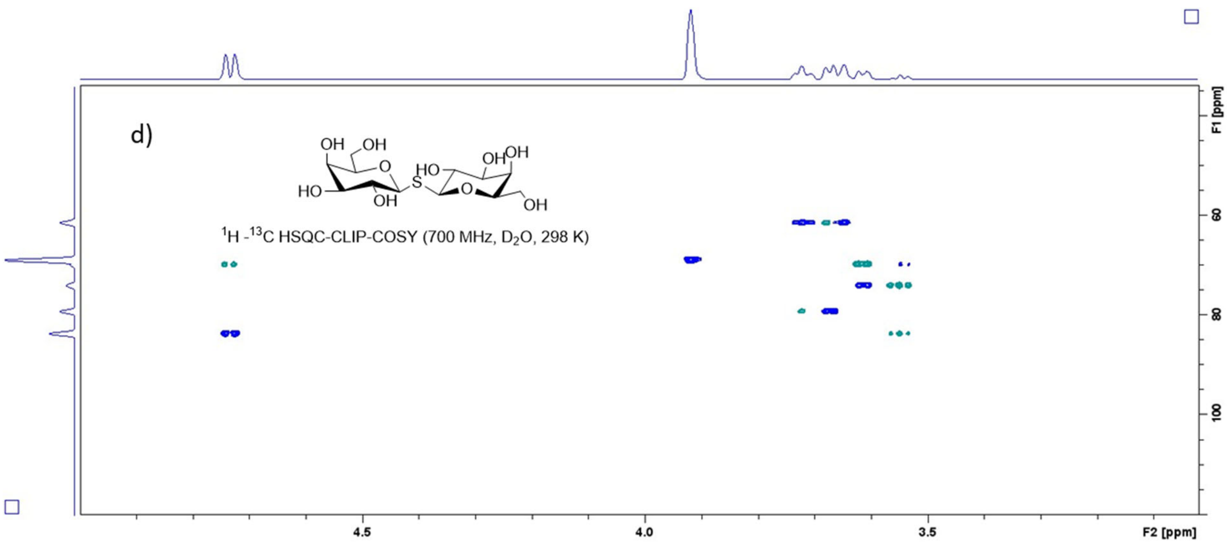

Figure S2. ^{1}H (a), *J*-modulated ^{13}C (b), ^{1}H-^{1}H COSY (c), ^{1}H-^{13}C HSQC (d), ^{1}H-^{13}C HMBC (e), ^{1}H-^{13}C HSQC-CLIP-COSY (f) NMR spectra of (**1**)

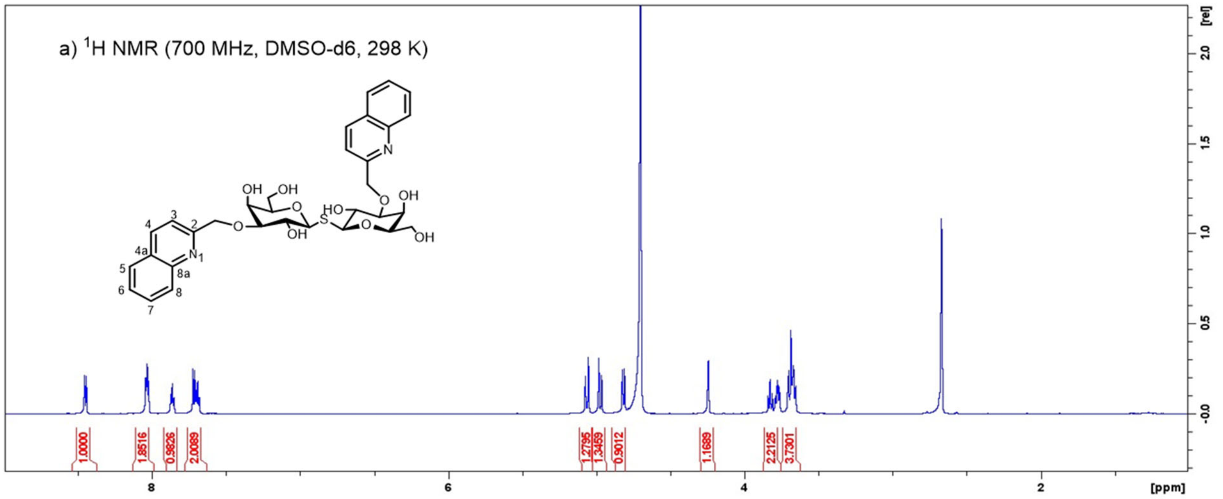

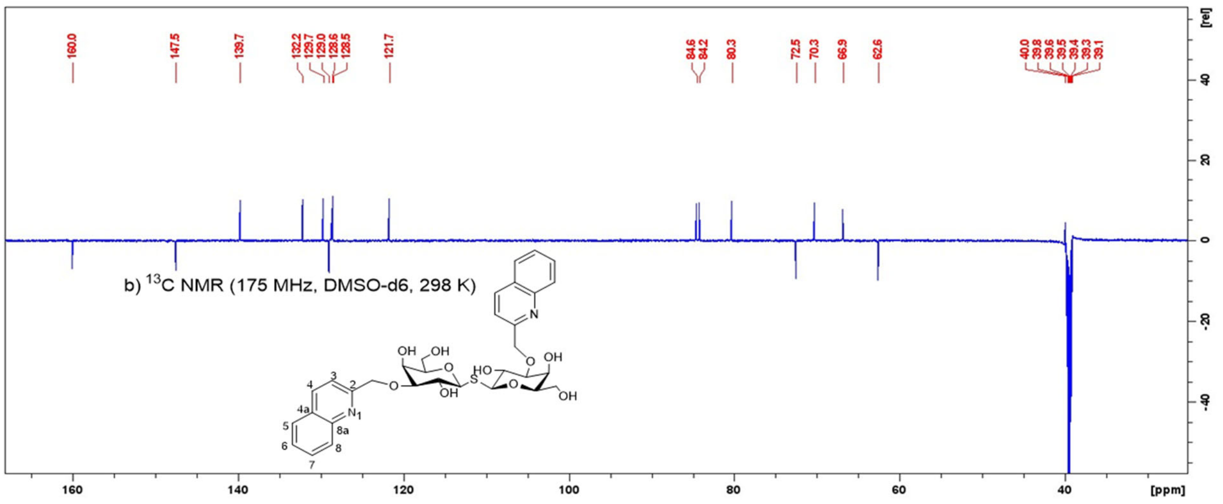

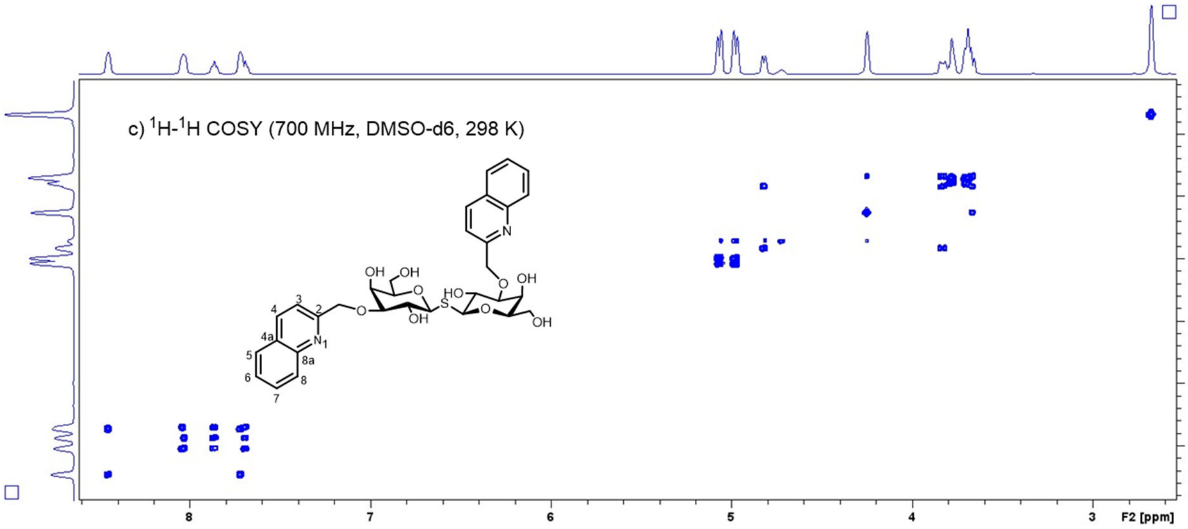
c) 1H-1H COSY (700 MHz, DMSO-d6, 298 K)
F1 [ppm]
F2 [ppm]

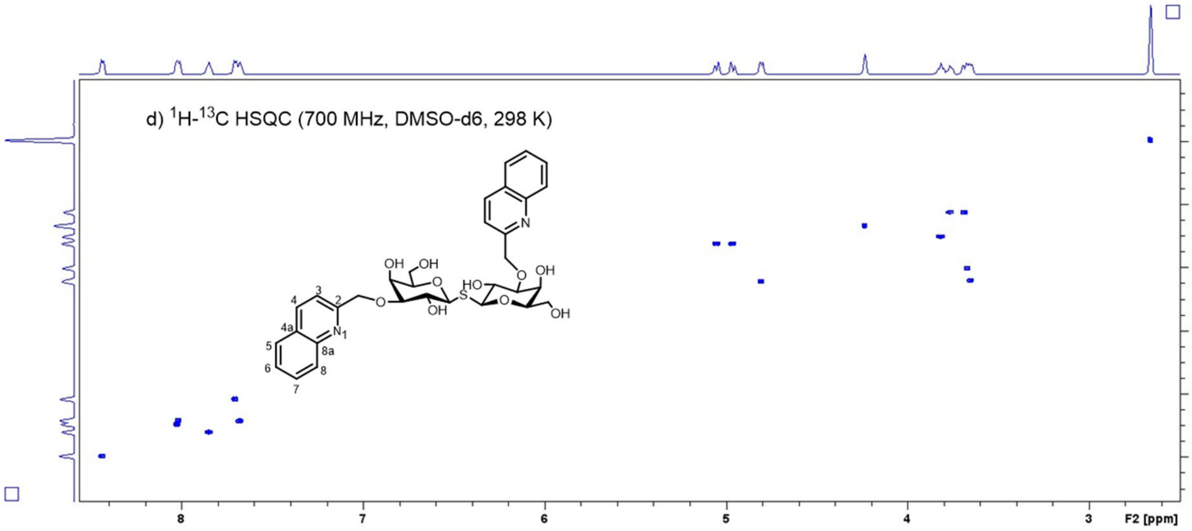
d) 1H-13C HSQC (700 MHz, DMSO-d6, 298 K)
F1 [ppm]
F2 [ppm]

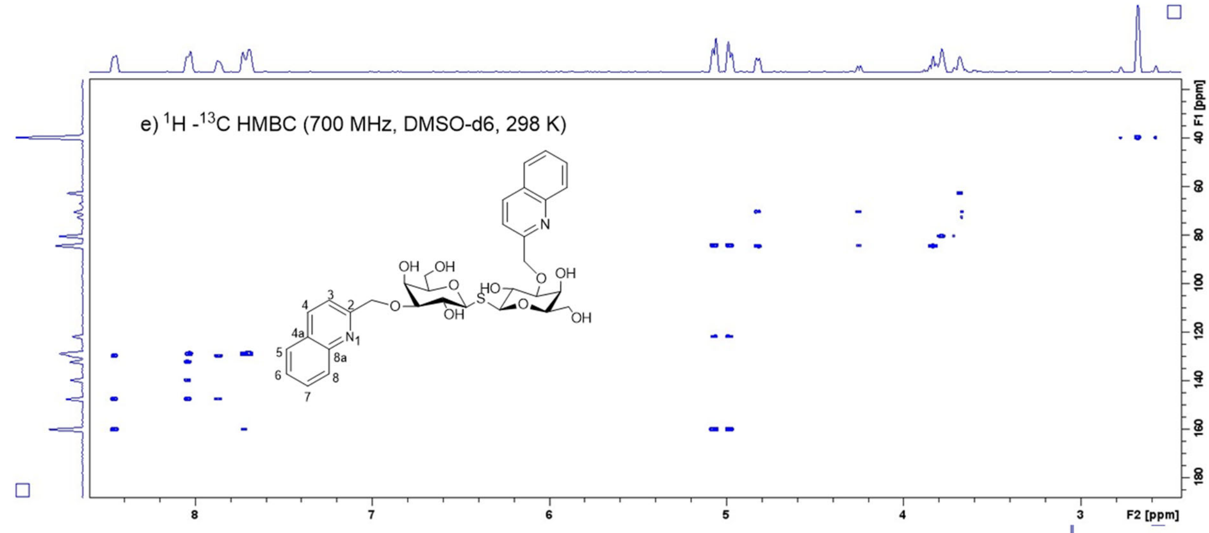
e) ^{1}H -^{13}C HMBC (700 MHz, DMSO-d6, 298 K)
F1 [ppm]
F2 [ppm]

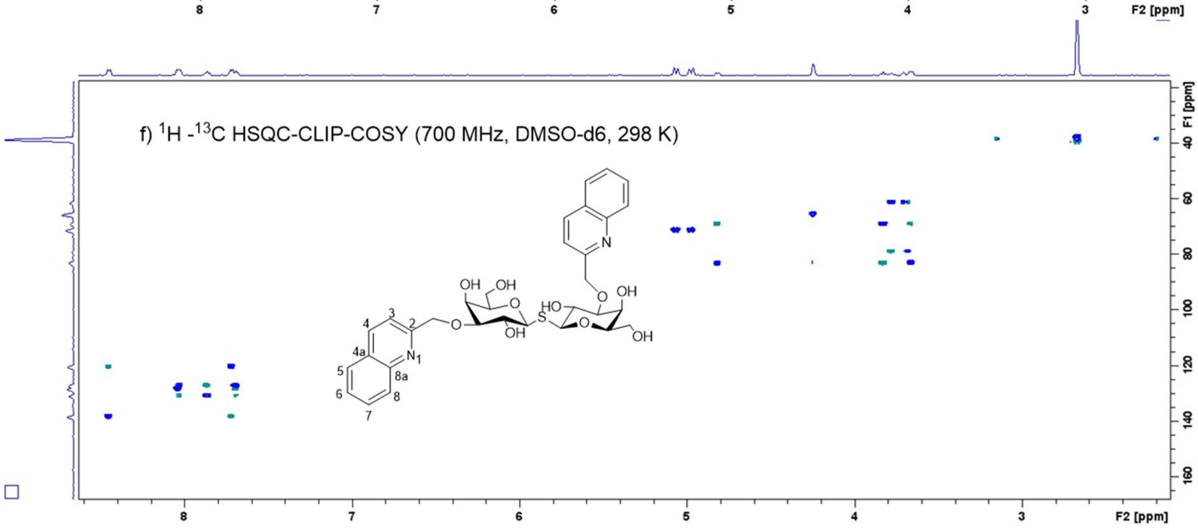
f) ^{1}H -^{13}C HSQC-CLIP-COSY (700 MHz, DMSO-d6, 298 K)
F1 [ppm]
F2 [ppm]

Figure S3. ^{1}H (a), *J*-modulated ^{13}C (b), ^{1}H-^{13}C COSY (c) ^{1}H-^{13}C HMBC (d), ^{1}H-^{13}C HSQC-CLIP-COSY (e) NMR spectra of (**2**)

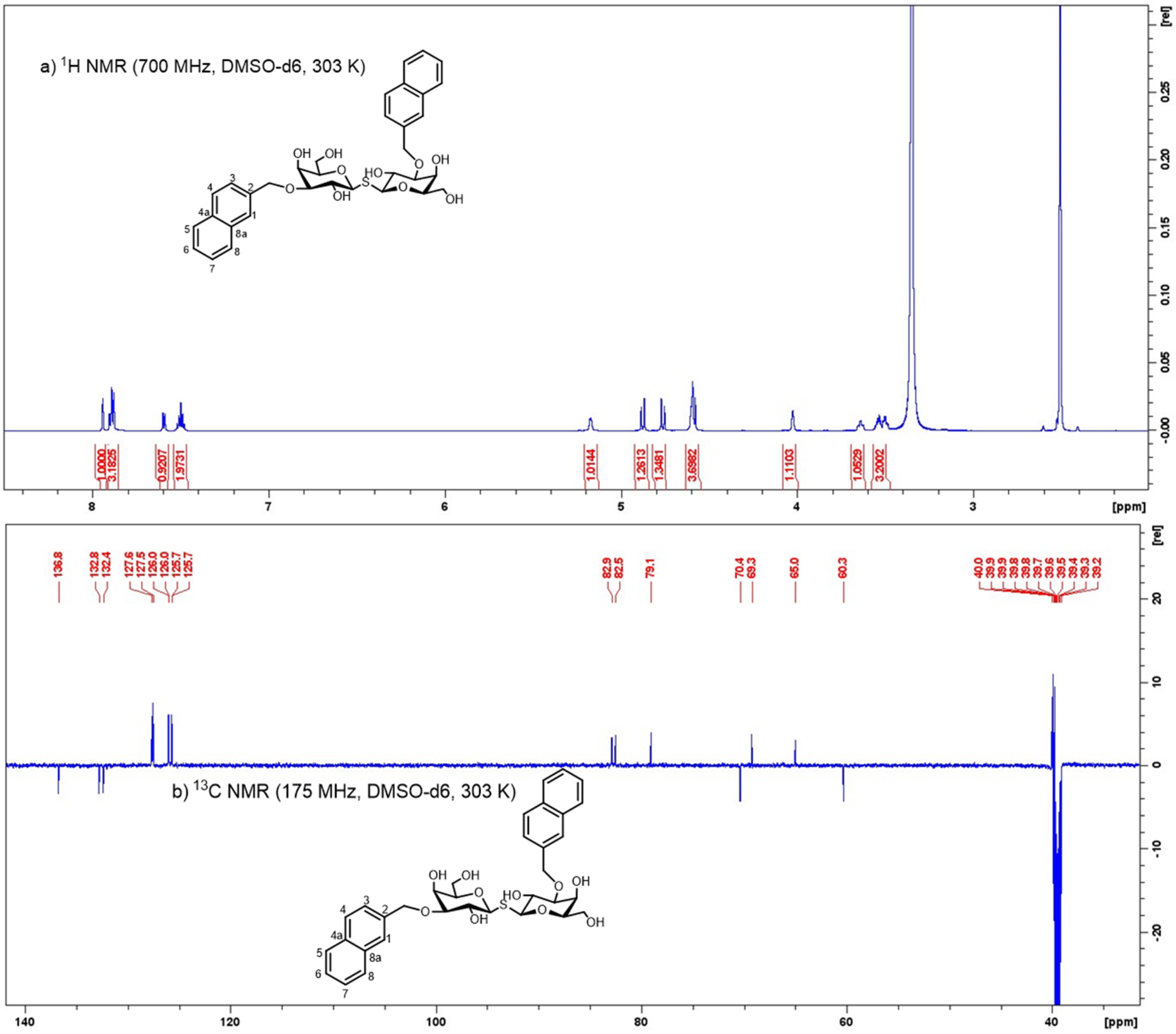

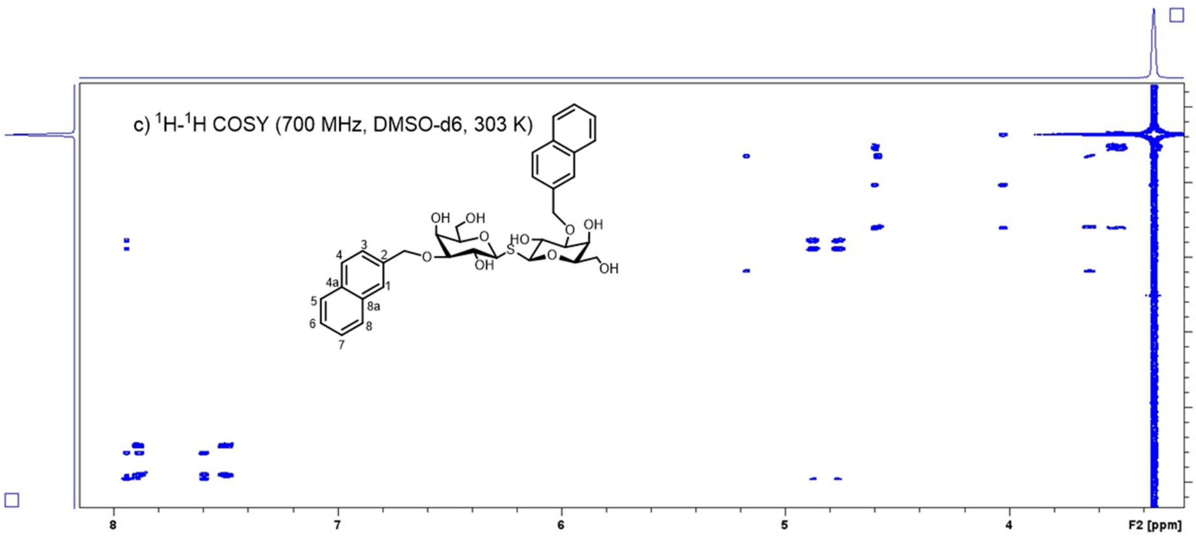
c) 1H-1H COSY (700 MHz, DMSO-d6, 303 K)
F1 [ppm]
F2 [ppm]

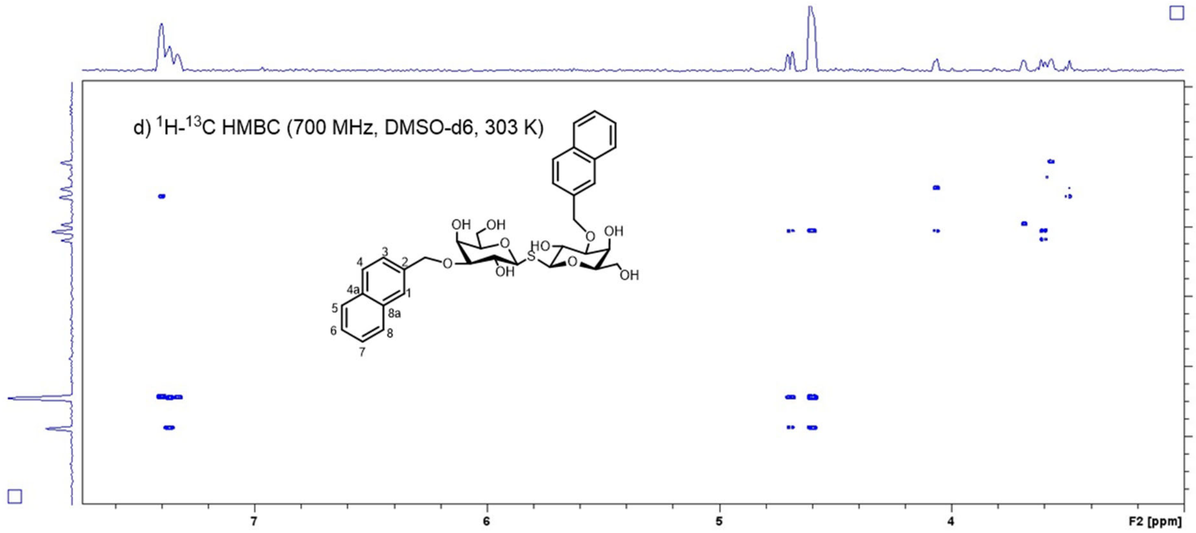
d) 1H-13C HMBC (700 MHz, DMSO-d6, 303 K)
F1 [ppm]
F2 [ppm]

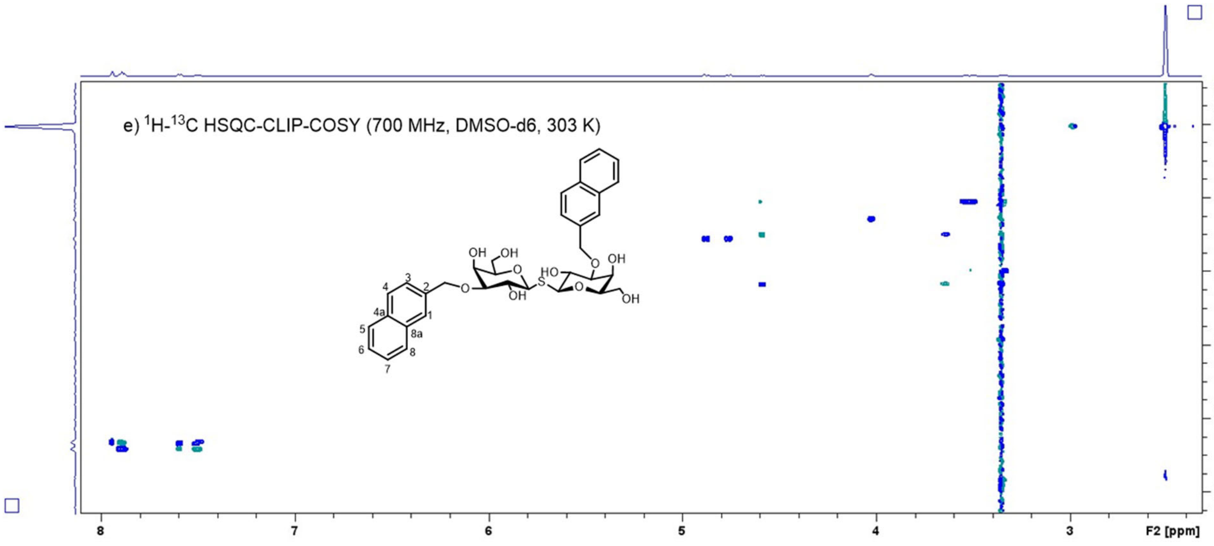
e) 1H-13C HSQC-CLIP-COSY (700 MHz, DMSO-d6, 303 K)
F1 [ppm]
F2 [ppm]

Figure S4. ^{1}H (a), *J*-modulated ^{13}C (b), ^{1}H-^{13}C HSQC (c) ^{1}H-^{13}C HMBC (d), ^{1}H-^{13}C HSQC-CLIP-COSY (e) spectra of (**3**)

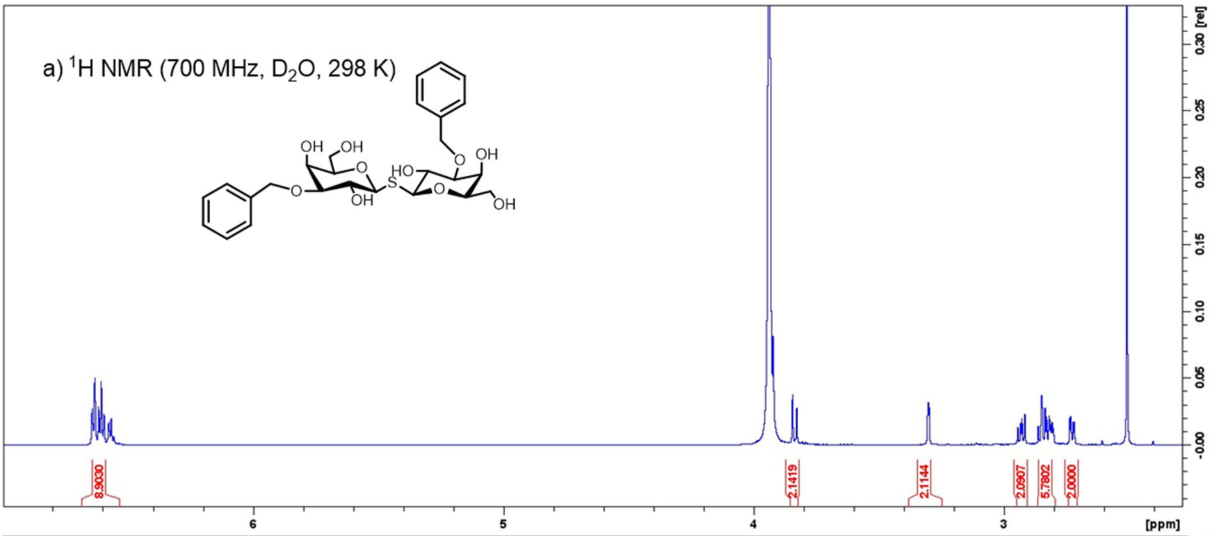

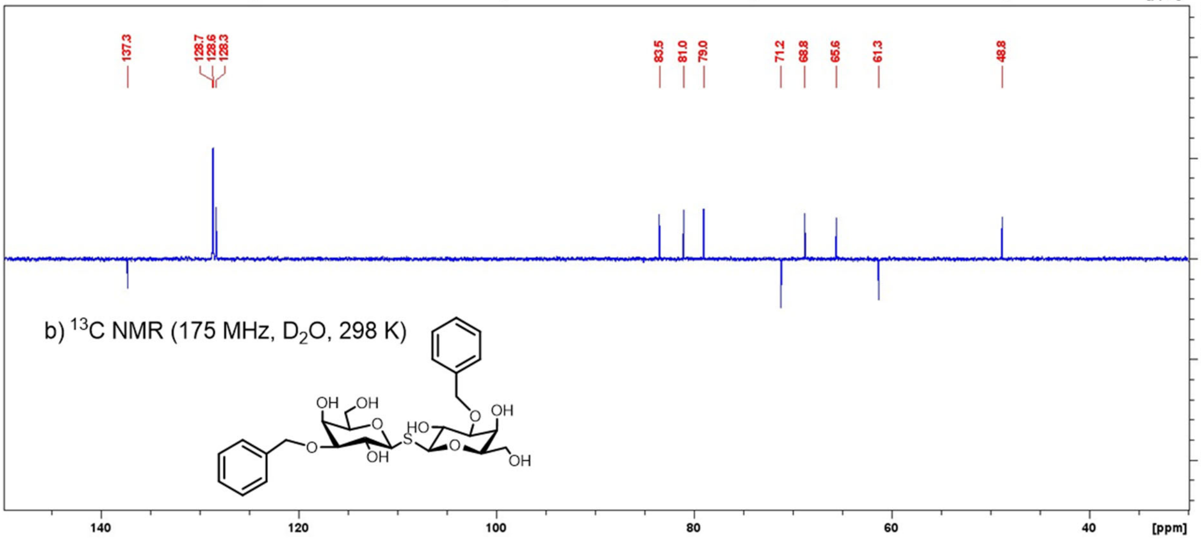

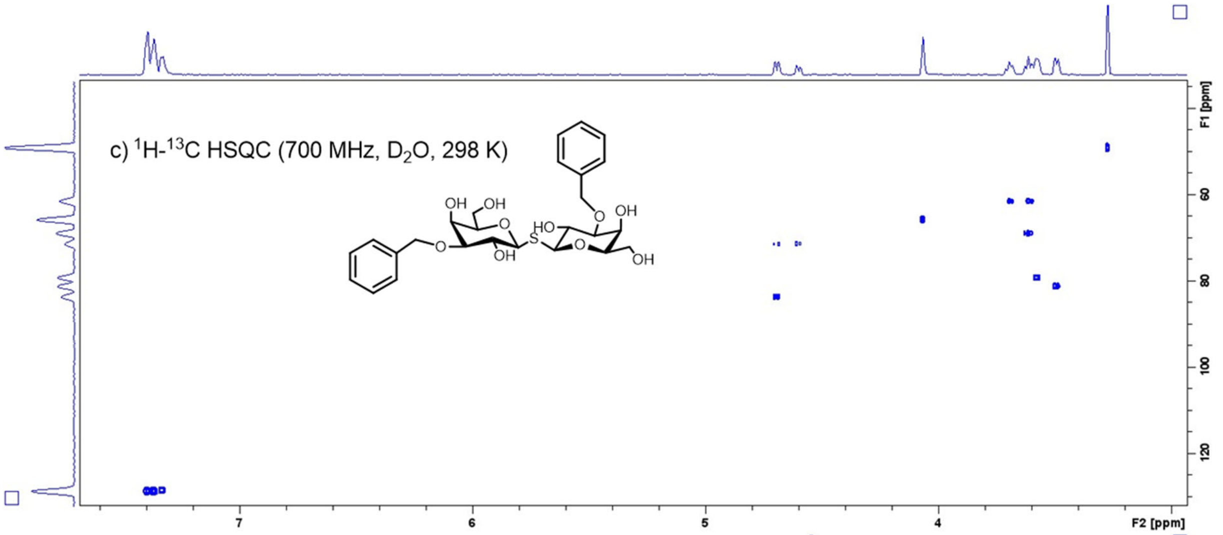
c) ^{1}H-^{13}C HSQC (700 MHz, D_2O, 298 K)
F1 [ppm]
F2 [ppm]

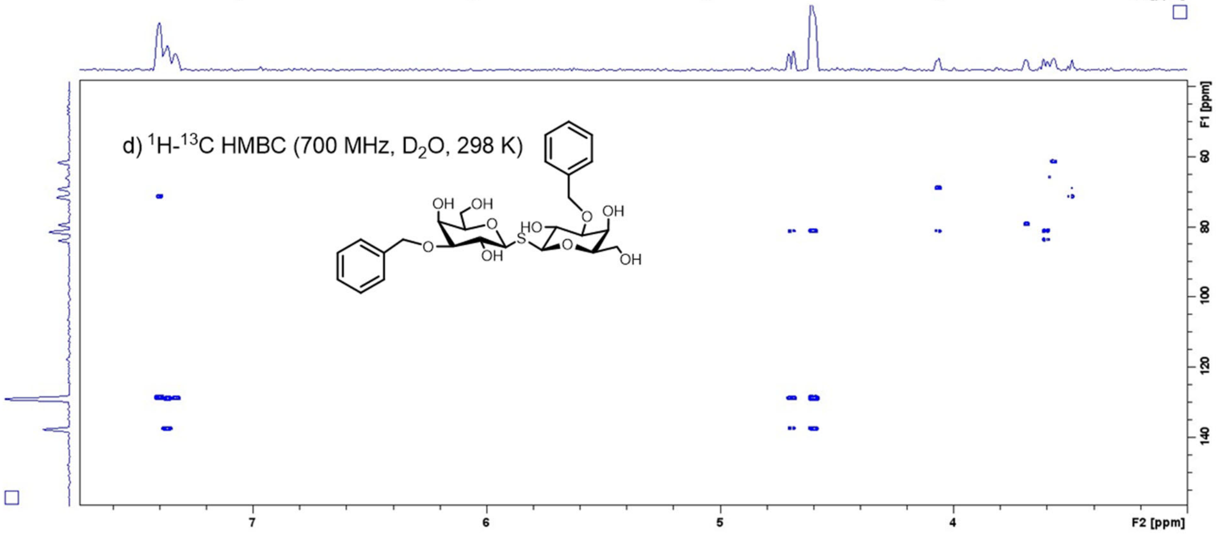
d) ^{1}H-^{13}C HMBC (700 MHz, D_2O, 298 K)
F1 [ppm]
F2 [ppm]

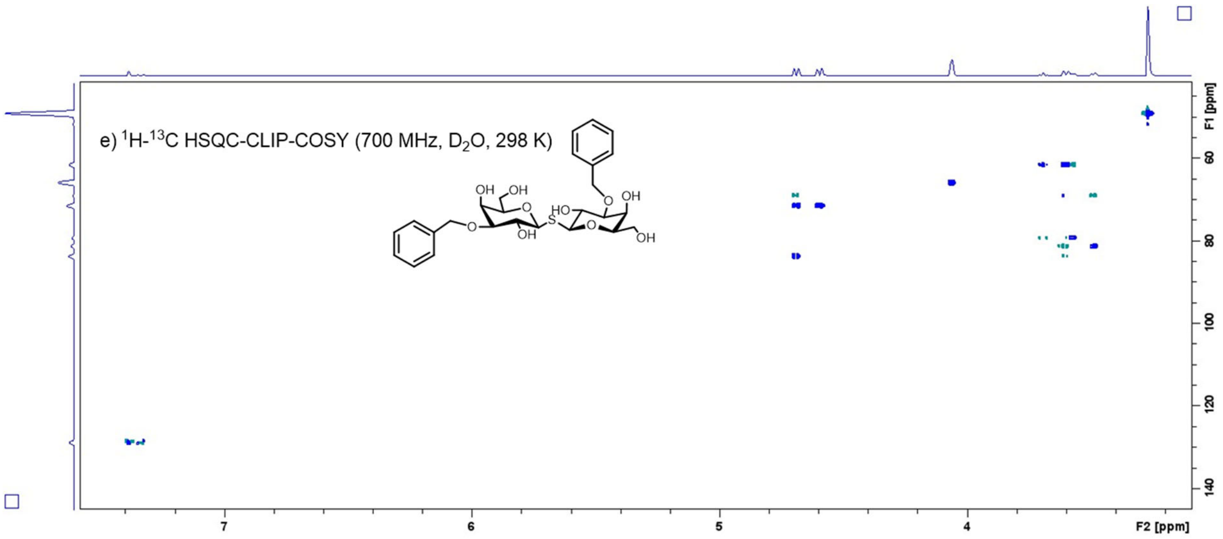
e) ^{1}H-^{13}C HSQC-CLIP-COSY (700 MHz, D_2O, 298 K)
F1 [ppm]
F2 [ppm]

Figure S5. ^{1}H (a), *J*-modulated ^{13}C (b), ^{1}H-^{1}H COSY (c), ^{1}H-^{13}C HSQC (d) NMR spectra of (**4**)

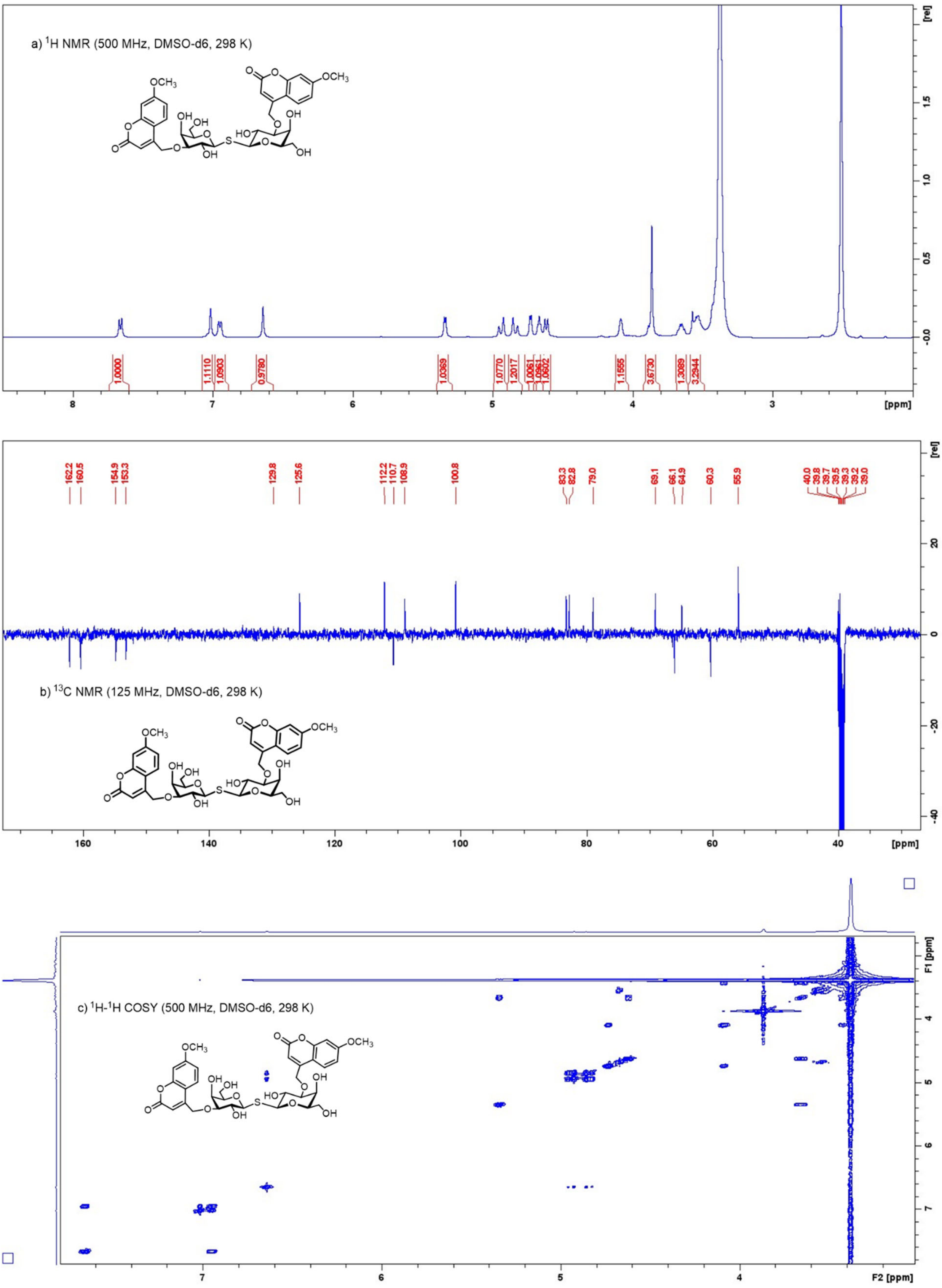

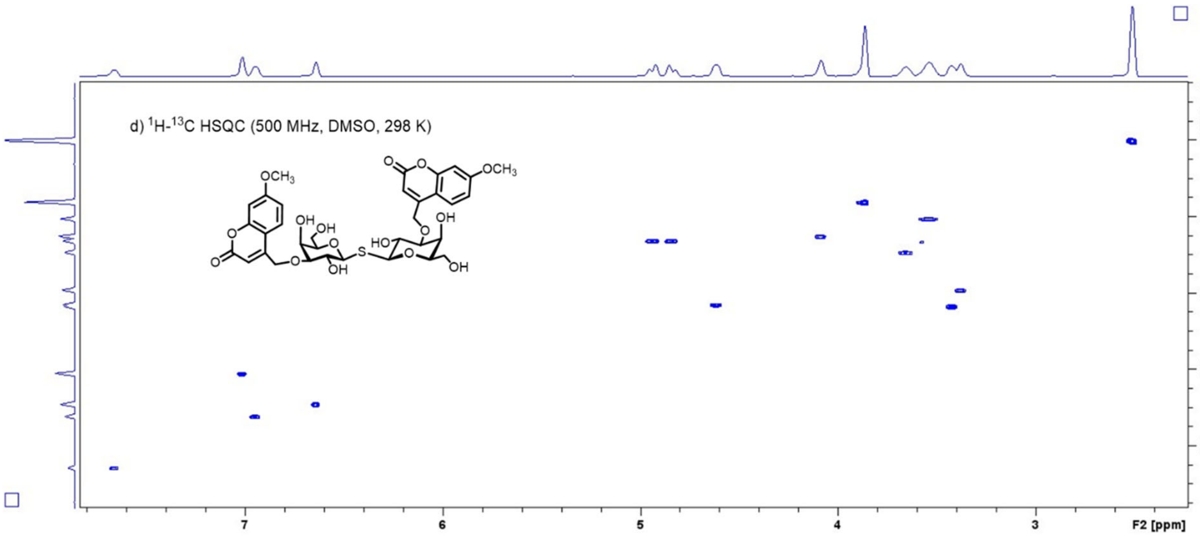

d) 1H-13C HSQC (500 MHz, DMSO, 298 K)
OCH3
OH
OH
O
O
O
O
S
HO
O
OH
O
OH
OH
O
O
OCH3
40 F1 [ppm]
60
80
100
120
7
6
5
4
3
F2 [ppm]
